# Supplementary material for: Systematic review of clinical outcome reporting in randomised controlled trials of burn care
Source: BMJ Open. 2019 Feb 15;9(2):e025135. doi: 10.1136/bmjopen-2018-025135 (PMC6398699; doi:10.1136/bmjopen-2018-025135)
Supplement: Author's manuscript [file bmjopen-2018-025135.draft_revisions.pdf]

BMJ Open

BMJ Open is committed to open peer review. As part of this commitment we make the peer review history of every article we publish publicly available.

When an article is published we post the peer reviewers' comments and the authors' responses online. We also post the versions of the paper that were used during peer review. These are the versions that the peer review comments apply to.

The versions of the paper that follow are the versions that were submitted during the peer review process. They are not the versions of record or the final published versions. They should not be cited or distributed as the published version of this manuscript.

BMJ Open is an open access journal and the full, final, typeset and author-corrected version of record of the manuscript is available on our site with no access controls, subscription charges or pay-per-view fees (http://bmjopen.bmj.com).

If you have any questions on BMJ Open's open peer review process please email info.bmjopen@bmj.com

BMJ Open

A systematic review of clinical outcome reporting in randomised controlled trials of burn care.

Journal:	*BMJ Open*
Manuscript ID	bmjopen-2018-025135
Article Type:	Research
Date Submitted by the Author:	04-Jul-2018
Complete List of Authors:	Young, Amber; University Hospitals Bristol NHS Foundation Trust, Paediatric Anaesthesia; University of Bristol, Canynge Hall<br>Davies, Anna; University of Bristol, Population Health Sciences<br>Bland, Sophie; West of England Academic Health Science Network<br>Brookes, Sara; Institute of Cancer and Genomic Sciences, University of Birmingham, Cancer Research UK Clinical Trials Unit ; University of Bristol, Population Health Sciences<br>Blazeby, Jane; University of Bristol, Population Health Sciences
Keywords:	Clinical decision-making, Review, Treatment Outcome, Outcome Assessment (Health Care), Burns, Randomised Controlled Trials as Topic

SCHOLARONE™ Manuscripts

For peer review only

A systematic review of clinical outcome reporting in randomised controlled trials of burn care.

Amber E Young[1], Anna Davies[1], Sophie Bland[2], Sara Brookes[1,3], Jane M Blazeby[1]

[1]Population Health Sciences, Bristol Medical School, University of Bristol, Canynge Hall, Bristol, UK

[2]Patient representative

[3]Cancer Research UK Clinical Trials Unit (CRCTU), Institute of Cancer and Genomic Sciences, University of Birmingham, Edgbaston, Birmingham, UK

Amber.young1@nhs.net

Anna.davies@bristol.ac.uk

Sophie.bland@weahsn.net

s.t.brookes@bham.ac.uk

j.m.blazeby@bristol.ac.uk

Abstract

Systematic reviews collate trial data to provide evidence to support clinical decision-making. For effective synthesis, there must be consistency in outcome reporting. There is no set of agreed outcomes for reporting the effect of burn care interventions. Issues with outcome reporting have been previously identified although not systematically investigated. This study gathers empirical evidence on the extent of the problem and ascertains the need for a Core Outcome Set for burn care research.

Methods: Electronic searches of Ovid MEDLINE, Ovid EMBASE, Web of Science and The Cochrane Library were undertaken from January 2012 to December 2016 for RCTs using medical subject heading and free text terms including 'burn', 'scald' 'thermal injury' and 'RCT'. Two authors independently screened papers, extracted outcomes verbatim and recorded the timing of outcome measurement. Duplicate outcomes (exact wording +/- different spelling), similar outcomes (albumin in blood, serum albumin) and identical outcomes measured at different times were removed. The number of unique outcomes in each trial was recorded. Variation in outcome reporting was assessed across trials. Outcomes were classified into domains.

Results: 147 trials were included, of which 127 (86.4%) were RCTs, 13 (8.8%) pilot studies and 7 (4.8%) RCT protocols. 1,494 verbatim clinical outcomes were reported; of these, 955 were unique. 76.8% of outcomes were measured within six months of injury and 11.7% after one year. Numbers of unique outcomes per trial varied

For peer review only - http://bmjopen.bmj.com/site/about/guidelines.xhtml

from one to 37 (median 9; IQR 8). No outcome was reported in all studies. Outcomes were classified into 54 domains.

Conclusions: This review has demonstrated heterogeneity in selection, definitions and timing of outcome reporting in burn care trials. This will hinder amalgamation of trial results and limit evidence to support clinical decision-making. We recommend the development of a Core Outcome Set to standardise outcome reporting in burn care research.

Key words: Clinical decision-making, Review, Treatment Outcome, Outcome Assessment (Health Care), Burns, Randomized Controlled Trials as Topic

Strengths and limitations of this study:

- This review is a comprehensive and systematic search for all clinical outcomes reported in RCTs of burn care between 2012 and 2016.
- There is a detailed analysis of all reported outcomes and timing of outcome assessment.
- A multi-disciplinary team including a patient were involved in the study.
- Quality assessment of studies was not undertaken as the purpose of the review was to extract clinical outcomes.

For peer review only - http://bmjopen.bmj.com/site/about/guidelines.xhtml

For peer review only

Introduction

Each year an estimated 2-300,000 people die from burn injuries globally(1). Millions more suffer from burn-related disabilities and disfigurements(2). These injuries have functional, psychological, social and economic effects on survivors and their families. There are multiple strategies for managing burn wounds and associated impact on patient physiology, with new care pathways and technology being introduced on a regular basis(3-5). The choice of treatment should be made using up-to-date, high quality scientific evidence (6, 7). Systematic reviews of randomised controlled trials (RCT) are regarded as the highest quality evidence (8-10). Despite increasing numbers of published randomised controlled trials (RCTs) in burn care, systematic reviews have not provided evidence to support many commonly used interventions or management strategies (11-13).

A well-designed RCT requires that outcomes are pre-specified. Evidence synthesis requires that these outcomes are consistent across RCTs in the same healthcare area (14). In the context of clinical trials, Williamson et al in the Core Outcome Measures in Effectiveness Trials (COMET) handbook, define an outcome as "a measurement or observation used to capture and assess the effect of treatment such as assessment of side effects (risk) or effectiveness (benefits)" (15). Chan adds a temporal element: "a variable measured at a specific time point to assess the efficacy or harm of an intervention"(16). If RCTs report outcomes that cannot be collated due to differences in choice, definition or timepoint of assessment, evidence synthesis will not be effective or efficient. There is no agreed minimum set of outcomes important to patients and professionals for reporting in burn care trials and problems with outcome reporting in burn care research have previously been suggested(17-19).

Pre-specifying outcomes requires research to determine and agree the most important outcomes for a clinical condition. If this is not undertaken, the outcomes reported may not reflect patients' or other stakeholders' needs, outcomes will vary between studies (outcome reporting heterogeneity) and it will be difficult to determine if authors have reported all the outcomes they measured (outcome reporting bias) (20, 21). Choosing the most important outcomes to measure in burn care is complex, as patients are a heterogeneous population, with variations in age, mechanism of injury, depth, site and size of burn (22, 23). The time frame at which outcomes are measured may also determine the types of outcomes assessed. Outcomes reported in clinical trials during the acute treatment phase include healing time, skin-graft loss, infection rates and NHS costs (24-27). Longer-term reported outcomes relate to functional, cosmetic and psychological issues (28).

To date, there has been no formal investigation into outcome reporting in trials of burn care. The purpose of this study is to examine clinical outcome reporting in burn care research, to consider the types, definitions and timing of outcomes measured and to consider the need for a Core Outcome Set in this field.

For peer review only - http://bmjopen.bmj.com/site/about/guidelines.xhtml

Methods

This review is focused on clinical observer-assessed outcomes reported in RCTs assessing the impact of interventions in burn care. It adhered to a pre-specified protocol and the Preferred Reporting Items for Systematic Reviews and Meta-Analyses (PRISMA) statement(29). It was registered with the PROSPERO international prospective register of systematic reviews (ID CRD42017060908).

Study Eligibility

Studies were included if they met the following:

Types of studies: We included full text RCTs along with RCT protocols and pilot studies. The study design was limited to RCTs, as a final core outcome set will be used for RCT reporting(29). We excluded protocols and pilot studies if the full RCT had been published. We also excluded conference proceedings and abstracts, non-English language publications and studies not involving human subjects.

Types of participants: We included studies recording outcomes from patients of any age with a cutaneous burn of any type or size, determined by either clinician evaluation or objective assessment, or both, which required treatment in any health care facility. Studies where the population consisted of patients with combined thermal and mechanical injuries were only included if it was possible to separate out the burn care outcomes. Trials studying patients with pure carbon monoxide poisoning, chemical ocular or caustic oesophageal burns were excluded, as the former does not involve a burn and the latter have different aetiology and management to cutaneous burns.

Type of interventions: Any surgical or non-surgical burn care intervention with any appropriate *comparator.*

Types of outcomes: defined as the exact terms used in a published trial Abstract, Methods or Results including tables and figures for any observer-reported clinical endpoint. These included physiological, metabolic or adverse or mortality events measured by researchers and relevant to patients' recovery and long-term well-being after burn care (30). Trials assessing quality of life were only included if the data were observer-reported.

Identification of studies

Electronic searches of Ovid MEDLINE, Ovid EMBASE, Web of Science and The Cochrane Library were searched from 1st January 2012 to December 31st 2016 for RCTs related to burn care using medical subject heading and free text terms including 'burn', 'scald' 'thermal injury' and 'RCT'. This period was chosen so that the outcomes extracted reflected use in trials relating to modern burn care. The thesaurus vocabulary of each database was

For peer review only - http://bmjopen.bmj.com/site/about/guidelines.xhtml

used to adapt the search terms. The search strategy for Ovid MEDLINE is included in a previous publication and in Appendix A(29).

Cohen's Kappa was calculated to report inter-rater reliability of selection at full text stage for both inclusion and reason for exclusion, corrected for chance agreement (28). Data were managed in a Microsoft Excel database.

Study selection process

Prior to both abstract and full-text screening, all review authors underwent training to ensure a comparable understanding of the purpose of the review and the eligibility criteria. The reference management software EndNote (Endnote 6, Clarivate Analytics, Boston; available at http://endnote.com/) was used to compile all titles derived from the initial searches, with duplicates removed for the review authors to screen titles and abstracts against the eligibility criteria. Screening of titles and abstracts was completed independently, then in duplicate by two authors (AY, AD) with experience in systematic review methodology. All screening disagreements were discussed, with any outstanding disagreements resolved by an independent reviewer (JB). Any studies appearing to meet the inclusion criteria based on the abstract were retrieved as full-text articles. Two reviewers then read the full-text articles in their entirety to assess for eligibility, with decisions on inclusion and exclusion recorded (Figure 1 for flow diagram). Reasons for exclusion were ordered hierarchically (Table 1) and applied to each full text. The highest reason for exclusion met by a paper was recorded as its reason for exclusion. Any disagreements were discussed with another author (JB).

Quality Assessment.

The aim of this study was to comprehensively document clinical outcomes recorded in burn care RCTs and not to synthesise data about the effect of interventions. An assessment of risk of bias of the included trials was therefore not undertaken.

Data Extraction

Data was extracted into a standardised data extraction sheet. This included study author, country or countries recruiting (categorised into the United Nations six regions (31)), publication year, number of sites and number of participants recruited per trial, design (full RCT, pilot, protocol) and intervention tested. For protocols, the planned participant inclusion criteria and sample size were extracted.

No distinction was made between primary or secondary outcomes, although this was noted and is part of a separate project. All outcomes were extracted verbatim, with 20% of the extracted data verified by a second reviewer. True duplicates, spelled and worded the same, were deleted. As a second process, two reviewers (a clinician and researcher) discussed all verbatim outcomes to assess duplicates in meaning but spelled or worded

For peer review only - http://bmjopen.bmj.com/site/about/guidelines.xhtml

in a slightly different manner; such as length of time in hospital and number of days in hospital, platelet level and levels, and serum IL-10 and IL-10 in blood. These were named as one outcome with wording chosen by the reviewers and the others deleted as duplicates. The remaining outcomes were therefore all different in meaning. Any discrepancies were discussed with a senior researcher (JB). The number of outcomes per trial and the variation of outcomes between trials was recorded.

The time after injury that outcomes were measured were noted separately in order to a) assess the heterogeneity in outcome measurement timing and b) to understand at what stage after injury the effects of the intervention were being assessed. If a single outcome was assessed at different timepoints, *all* assessment timings were recorded. Data extraction for the timing of outcome reporting from 10% of trials was undertaken independently by another researcher. Timings of outcome assessment were categorised into time periods; $\leq$ 1 month, > 1 month and $\leq$ 3 months, > 3 months and $\leq$ 6 months after injury, > 6 months and $\leq$ 1 year and > 1 year and $\leq$ 3 years, and > 3 years. We reported two other outcome time periods; those assessed during acute hospitalisation and during burn wound healing, as these were commonly reported in the literature with no proscribed timepoint. However, it was clear from the reported length of stay and healing data, that all these outcomes were assessed within six months of injury. The frequency of outcomes reported within each time period was recorded.

The data were tabulated in a Microsoft excel spreadsheet, so that each study was listed with study and population details along with outcomes measured. Outcomes were extracted from this spreadsheet into another, with duplicates removed as described above. Outcomes measuring the same healthcare issue but at different timepoints were noted as one outcome for the final set. These final unique outcomes were then grouped into domains.

Classification of outcomes into domains

Outcome domains are groups of similar outcomes. This organisation is necessary, as maintaining a large set of outcomes when a significant number are similar would make any future classification of the outcomes in terms of importance, extremely challenging.

Outcomes were classified into domains in three stages. In stage one, three researchers (two clinically trained burns researchers, one lay burn researcher) independently reviewed the list of outcomes and attributed a domain to each one using their own terms. In stage two, the researchers met to review the domains and agreed i) an appropriate name for each domain, and ii) the appropriate domain for each outcome. Rules for attribution of outcomes to domains were recorded in a coding log to ensure consistency. In stage three, a fourth lay researcher and a patient representative (SB) reviewed the outcomes and their attributed domains to check for

For peer review only - http://bmjopen.bmj.com/site/about/guidelines.xhtml

clarity of domain name, and that the outcomes under each domain were appropriately attributed. A final meeting with an experienced outcome researcher (JB) was held to finalise outcomes and domains.

The results described below indicate the characteristics of the reported studies and provide detail on heterogeneity of outcome reporting between studies, outcome timepoints and outcome domains.

Patient Involvement: The need for a burn care Core Outcome Set project was conceived following discussions regarding clinical healthcare Key Performance Indicators with professionals and patients. The patients were vocal about outcomes important to them which they felt were overlooked by professionals, such as pain. The systematic review was discussed at regular project steering group meetings attended by three patients with burns and one parent of a child with burns. A patient with burns is a co-author and was involved with writing and editing of this article as well as with the naming of the outcome domains. Dissemination will be to the lay representatives of the steering group and will inform the Core Outcome Set study in which patients are actively involved.

Results

Included studies and study protocols:

The initial search strategy identified 3,110 studies. Following de-duplication, a total of 2,070 studies remained. Independent scrutiny of the titles and abstracts identified 306 potentially relevant articles for full text review. Of these, 158 studies did not meet our inclusion criteria and were excluded (PRISMA Flow Diagram Figure 1). Therefore, a total of 147 studies formed the basis of this study(24, 32-176).

Reliability of study selection: Cohen's Kappa was calculated to report inter-rater reliability of selection at full text stage for both inclusion and reason for exclusion. The results were 0.751 and 0.747 respectively. This is rated as substantial agreement.

Studies: Of the 147 studies, 86.4% (127) were reports of full RCTs, 8.8% (13) were pilot studies and 4.8% (7) were study protocols. The number of studies published increased between 2012 and 2016, with 26 RCTs published in 2012 and 40 in 2016 (Table 2).

9,022 participants were recruited across the 140 studies (study protocols not included n=7). The number of patients recruited per trial ranged from 3 to 612 (median 50; IQR 30-88) for full RCTs and from 10 to 52 (median 21; IQR 16-28) for pilot studies. 50.4% of full RCTs recruited fewer than 50 participants (Table 2). The majority (89.7%) of studies recruited (or planned to recruit) participants on one site alone. Of the 10.2% (15) of studies that were multi-centre, nine (60%) undertook research at only two or three sites. Thirty-two countries from the

For peer review only - http://bmjopen.bmj.com/site/about/guidelines.xhtml

six global regions recruited patients into the 147 RCTs (Table 2). The country that undertook the most studies was the USA with 22.4% (33), followed by Iran with 12.9% (19) and China with 10.9% (16) of published studies. Of the 32 countries, 59.3% (19) published only one trial in this time period. The most common trial interventions related to dressings and wound care 29.2% (43), followed by surgical technique 11.6% (17) and management of pain and itch 10.9% (Table 2).

Outcomes:

1,494 clinical outcomes were reported of which, after de-duplication, 955 different outcomes remained. Of the 1,494 outcomes reported, 27.7% (421) were common across two studies or more. Of *these* outcomes, 50.3% (78) appear in only two trials and 84.5% appear in five trials or fewer. No single outcome was reported across all 147 studies. The number of outcomes reported per trial varied from one to 37 (median 9; IQR 8) (Table 3). Outcomes assessing the same healthcare issue were commonly defined differently. An example is burn wound healing which was defined in 170 different ways. Examples include: healing percentage at specified timepoints, incidence of complete wound healing, incidence of 30% wound healing and length of time until 50% epithelialisation of burn wound.

Outcome timing: There were 2,743 outcomes measured if the same outcome measured at different timepoints across all the 147 RCTs are included; e.g. size of burn wound measured at one week and again at two weeks, were recorded as different outcomes for this exercise. Of these, 76.9% (2,109) were assessed at less than six months after injury, 16.6% (456) were measured after six months and before three years after injury, and only 5.1% (140) were measured at more than three years after injury (Figure 2). The timing of outcome measurement was not reported for 38 outcomes.

Outcome domains: The 990 *different* clinical outcomes were organised into 54 domains (groups of similar outcomes) (Table 4 and Appendix B).

Discussion

This systematic review aimed to examine outcome reporting in RCTs in burn care. Of the 147 included studies, 1,494 outcomes were identified, with overlap in terminology, inconsistent definitions and variation in time after injury at which the outcomes were measured. Only 30% of the outcomes reported were included in more than one study. There was no single outcome reported in all 147 trials. 77% of outcomes were measured at less than six months after injury. Such heterogeneity of outcome reporting across trials will limit evidence synthesis and mean that research is wasted.

For peer review only - http://bmjopen.bmj.com/site/about/guidelines.xhtml

The findings in this review have been seen elsewhere in the burns-specific and other clinical literature. A Cochrane review of 30 RCTs concluded that it was impossible to draw conclusions about burn dressing effectiveness, as the trials evaluated a variety of clinical outcomes(18, 177). Over the same period as this review, nine Cochrane reviews have had direct relevance to the management of patients with burns(18, 178-185). None could draw firm conclusions due to methodological issues including heterogeneity of outcome reporting. Heterogeneity is found in the reporting of outcomes relating to critical care, neurological illness, breast reconstruction surgery, prostate cancer, hip and knee replacement, oesophagectomy surgery, low back pain and in cardiac arrest trials among others (186-193). Variation in the definitions of outcomes has also been found within published studies of other healthcare areas. A systematic review of 90 papers reporting wound infection after general surgical procedures identified 41 definitions for wound infection itself, including three published by expert groups (194). Similarly, a total of 56 definitions were identified from 97 studies reporting anastomotic leak rates after gastrointestinal surgery despite publication of a standard definition two years before the beginning of the review (195). In this review we identified and agreed the grouping of 990 unique outcomes into 54 outcome domains and it is not always clear how to best categorise outcomes. Williamson published a taxonomy of categorising outcome domains (196) and others have suggested ways of doing this (210). The different approaches demonstrate a lack of agreement on how to optimally categorise outcomes. In burn care the large number and type of clinical domains identified and the variation in timing of assessments further complicates optimal domain classification underlining the need for further work in this area.

A solution to this variation in outcome reporting across trials, is the development of a Core Outcome Set (COS)(21, 197, 198). A COS is a minimum set of the most important outcomes, agreed and recommended for measurement in all trials for a particular condition (31, 32). While not limiting choice, a COS will pre-specify a set of outcomes to ensure consistency of reporting and the ability to collate evidence into systematic reviews by allowing researchers to compare 'like with like' (33). Trials can still select additional outcomes and primary outcomes in addition to the minimum core set. This approach has been shown to improve the consistency of outcome reporting (199, 200). Although there is no COS for burn care, work was undertaken in 2008 to agree a set of burn outcome domains(196). However, the work was undertaken by a small group of clinicians, lacked patient involvement and reported little methodological detail (201). Considerable work to develop COS methodology has also been undertaken since this publication (202, 203). The COMET (Core Outcome Measures in Effectiveness Trials) Initiative disseminates resources for COS development and supports methodological developments in this area(204, 205). COMET recommends a four-step process to develop a COS: a) agreement of the scope, b) assessment of the need, c) development of a protocol and finally d) agreement of the Core Outcome Set(15). This systematic review has satisfied the first two phases for the development of a burn care COS. The final phase encompasses organising a comprehensive long-list of all potential outcomes into domains (of which the clinical domains for burn care are listed in Table 4) and prioritising these domains using a consensus process (206-208).

For peer review only - http://bmjopen.bmj.com/site/about/guidelines.xhtml

The strengths of this review are that the protocol and data extraction proforma were pre-specified and the literature search was systematic and comprehensive, including four major healthcare trial databases. To account for multi-disciplinary perspectives, two researchers, two clinicians and a patient were involved in the domain process. It is also novel because it is the first to demonstrate, in detail and using systematic methodology, the scale of the heterogeneity of outcome reporting in burn care research. Limitations include the exclusion of publications in languages other than English. However, international publications were included to reduce the risk of selection bias. The search was also time-limited which may have excluded outcomes from older studies. The reason for the time limitation was to identify research relevant to modern burn care. The search was also limited to trials reporting clinical outcomes. Other work is in progress to assess patient-reported outcomes in burn care research. This was a review undertaken systematically to a pre-specified protocol. However, a formal quality assessment of studies was not undertaken, as we were researching the reporting of outcomes and not attempting to analyse the effects of interventions. A COS for burn care research would address the issue of heterogeneity of outcome reporting between trials, lead to research that is more likely to measure relevant outcomes, enhance the value of burn care systematic reviews and reduce research waste.

Conclusion

We have shown that multiple different outcomes are reported in trials of burn care interventions. Different definitions are used to assess the same outcome and outcomes are measured at different time points after injury. This heterogeneity and inconsistency in outcome reporting prevents effective evidence synthesis and limits the quality of evidence available for clinical decision-making. Our review demonstrates that until greater consistency is achieved in outcome reporting in trials, it is unlikely that clinicians will be able to synthesise evidence across studies to understand the effects of surgical and non-surgical treatments following burn injury. It is recommended that a burn care Core Outcome Set is developed to support the effective synthesis of trial data and allow more informed clinical decision-making for the benefit of patients.

Contributors: AY wrote the paper and conceived the project with the support of Professor JB. Professor JB and SB edited and critically revised the article. AD contributed to data extraction with support from JB and edited the paper. SBl assisted in the domain name choice and article editing and readability. All authors have read and approved the manuscript.

Acknowledgements: We would like to thank Dr Jason Wasiak, Senior Research Fellow, University of Melbourne, Monash University, Medical School, for his invaluable help in critically editing the paper. We would like to also thank Ms Joanna Hooper, senior outreach librarian and the University Hospitals Bristol NHS Foundation Trust medical library for their help in accessing articles.

For peer review only - http://bmjopen.bmj.com/site/about/guidelines.xhtml

Funding: This article/paper/report presents independent research funded by the National Institute for Health Research (NIHR) Doctoral Research Fellowship DRF-2016-09-031. The views expressed are those of the author(s) and not necessarily those of the NHS, the NIHR or the Department of Health. JB is part-funded by the Medical Research Council ConDuCT-II Hub (Collaboration and innovation for Difficult and Complex randomised controlled Trials In Invasive procedures—MR/K025643/1). The study was also supported by the NIHR Biomedical Research Centre at the University Hospitals Bristol NHS Foundation Trust and the University of Bristol.

PROSPERO number: ID CRD42017060908

Competing interests: JB is an NIHR Senior Investigator. All other authors declare no competing interests.

Ethics approval: South West Frenchay Research Ethics Committee (ref: 17/SW/0025
IRAS 221625).

Data Sharing: In regard to the data used as the basis for this study, a further Appendix (B) showing the outcome domains reported within each of the papers included in this systematic review, is available from the corresponding author.

For peer review only

For peer review only - http://bmjopen.bmj.com/site/about/guidelines.xhtml

Table 1. Hierarchy of exclusion

1. Duplicate
2. Not published between Jan 1st, 2012 until Dec 31st, 2016.
3. Not written in English
4. Not relating to burns or burn care alone.
5. Population is non-human subjects (e.g. animal studies, modelling)
6. Abstract with no full text given
7. Study is not an RCT testing an intervention on burn care
8. Laboratory based, not carried out in clinical setting e.g. exudate or blood samples from humans tested within laboratory.
9. Systematic review paper, commentary paper.
10. Reports only patient-reported outcomes
11. Volunteer study
12. Oesophageal burns
13. Ocular burns
14. Anaesthetic / sedation technique
15. Diagnostic test trial
16. Unavailable anywhere
17. Follow-up studies

For peer review only - http://bmjopen.bmj.com/site/about/guidelines.xhtml

Table 2: Randomised Clinical Trial detail

	Studies
Number of RCTs	127/147 (86.4%)
Number of Pilot studies	13/147 (8.8%)
Number of RCT protocols	7/147 (4.8%)
**World region for recruitment:**	
- Asia	54/147 (36.7%)
- North America	36/147 (24.5%)
- Europe	26/147 (17.7%)
- Africa	15/147 (8.5%)
- Latin America	1/147 (0.7%)
- Australasia	15/147 (8.5%)
**Year published:**	
- 2012	26/147 (17.8%)
- 2013	24/147 (16.3%)
- 2014	24/147 (16.3%)
- 2015	33/147 (22.4%)
- 2016	40/147 (27.2%)
**Number of sites:**	
- 1	132/147 (89.8%)
- 2-3	9/147 (6.1%)
- 4-5	2/147 (1.4%)
- > 5	4/147 (2.7%)
**Number of participants in full RCTs:**	
- ≤ 10	4/127 (3.1%)
- 11-50	62/127 (48.8%)
- 51-100	39/127 (30.7%)
- 101-150	11/127 (8.7%)
- >150	11/127 (8.7%)
**Participants recruited:**	
- ≤ 18 years	24/147 (16.3%)
- > 18 years	59/147 (40.1%)
- Mixed age range	25/147 (17.0%)
- Not stated	34/147 (23.1%)
- N/A (protocol)[c]	5/147 (3.4%)
**Type of intervention:**	
- Dressings and wound care	38/147 (25.9%)
- Surgical technique	19/147 (12.9%)
- Treatment of pain or itch[a]	16/147 (10.9%)
- Impact of exercise and rehabilitation	13/147 (8.8%)
- Intensive care management	10/147 (6.8%)
- Treatment of hypermetabolism	8/147 (5.4%)
- Nutrition	8/147 (5.4%)
- Scar management	7/147 (4.8%)
- Treatment of inhalational injury	3/147 (2.0%)
- Use of topical rHGM	3/147 (2.0%)
- Use of rHGH	3/147 (2.0%)
- Sugar management	2/148 (2.0%)
- Treatment of infection	2/147 (1.4%)
- Treatment of DVT	2/147 (1.4%)
- Blood management	2/147 (1.4%)
- Extracorporeal shock wave therapy	2/147 (1.4%)
- Platelet rich plasma use	2/147 (1.4%)
- Others[b]	7/147 (5.4%)

For peer review only - http://bmjopen.bmj.com/site/about/guidelines.xhtml

[a]Inc. distraction for dressing changes
[b]Inc. levamisole, hyperbaric oxygen, fibroblast growth factor, oral calcium use, ketoconazole, low intensity laser

Table 3: Reported outcomes

**Number of outcomes per study:**	
- 1	4/147 (27.2%)
- 2-5	34/147 (23.1%)
- 6-10	53/147 (36.1%)
- 11-20	41/147 (27.9%)
- >20	15/147 (10.2%)

Table 4: Outcome domains

Ability to carry out daily tasks	Generalised anxiety
Ability to fight infection	Growth in children
Anxiety about medical procedures and appointments	How well muscles work
Appearance	Infections other than burn wound infection
Blister fluid	Itch
Blood transfusion	Kidney function
Body weight maintenance	Length of hospital stay
Bone strength	Length of stay in intensive care unit
Breathing and lungs	Length of time on life support machine to aid breathing
Burden of care	Liver function
Burn wound healing	Medical tests to indicate how unwell a patient is
Burn wound infection	Mental ability
Burn wound pain	Mobility
Comfort of dressings	More than one organ failing
Complications of treatment	Muscle strength
Costs of treatment for NHS/Hospital	Pain during procedures
Death from burn injury	Psychological wellbeing
Death from any cause	Quality and quantity of sleep
Donor site healing	Return to work/school or previous function
Donor site pain	Scar colour
Donor site problems after healing	Scar pain
Effect of burn on genes	Scar texture
Effect of burn on how the body uses energy	Scar size
Effect of scar on movement	Sepsis
Effects of fluid from a drip	Stomach and bowel function
Effect on heart and blood circulation	Treatment for scars
Fitness	Use of medicines to treat symptoms

For peer review only - http://bmjopen.bmj.com/site/about/guidelines.xhtml

For peer review only

References:

1. Mock C, Peck M, Peden M, Krug E, Organization WH. A WHO plan for burn prevention and care. Geneva: World Health Organization. 2008:3.

2. Peck M, Molnar J, Swart D. A global plan for burn prevention and care. Bulletin of the World Health Organization. 2009;87(10):802-3.

3. Atiyeh BS, Hayek SN, Gunn SW. New technologies for burn wound closure and healing—review of the literature. Burns. 2005;31(8):944-56.

4. Jeschke MG, Shahrokhi S, Finnerty CC, Branski LK, Dibildox M, Organization A, et al. Wound coverage technologies in burn care: established techniques. Journal of Burn Care & Research. 2018;39(3):313-8.

5. Rowan MP, Cancio LC, Elster EA, Burmeister DM, Rose LF, Natesan S, et al. Burn wound healing and treatment: review and advancements. Critical care. 2015;19(1):243.

6. Sackett DL, Rosenberg WM, Gray JM, Haynes RB, Richardson WS. Evidence based medicine: what it is and what it isn't. British Medical Journal Publishing Group; 1996.

7. Dickinson HD. Evidence-based decision-making: an argumentative approach. International Journal of Medical Informatics. 1998;51(2-3):71-81.

8. Every‐Palmer S, Howick J. How evidence‐based medicine is failing due to biased trials and selective publication. Journal of evaluation in clinical practice. 2014;20(6):908-14.

9. Muir Gray J. Evidence-based healthcare: how to make health policy and management decisions. London: Churchill Livingstone. 1997;53.

10. Centre for evidence based medicine. CEBM Levels of evidence 2011 [Available from: https://www.cebm.net/wp-content/uploads/2014/06/CEBM-Levels-of-Evidence-2.1.pdf.

11. Flores O, Stockton K, Roberts JA, Muller MJ, Paratz JD. The efficacy and safety of adrenergic blockade after burn injury: A systematic review and meta-analysis. J Trauma Acute Care Surg. 2016;80(1):146-55.

12. Avni T, Levcovich A, Ad-El DD, Leibovici L, Paul M. Prophylactic antibiotics for burns patients: systematic review and meta-analysis. Bmj. 2010;340:c241.

13. Aziz Z, Abu S, Chong N. A systematic review of silver-containing dressings and topical silver agents (used with dressings) for burn wounds. Burns. 2012;38(3):307-18.

14. Liberati A, Altman DG, Tetzlaff J, Mulrow C, Gøtzsche PC, Ioannidis JP, et al. The PRISMA statement for reporting systematic reviews and meta-analyses of studies that evaluate health care interventions: explanation and elaboration. PLoS medicine. 2009;6(7):e1000100.

15. Williamson PR, Altman DG, Bagley H, Barnes KL, Blazeby JM, Brookes ST, et al. The COMET handbook: version 1.0. Trials. 2017;18(3):280.

16. Chan A-W, Krleža-Jerić K, Schmid I, Altman DG. Outcome reporting bias in randomized trials funded by the Canadian Institutes of Health Research. Canadian Medical Association Journal. 2004;171(7):735-40.

17. Van Baar M, Essink-Bot M-L, Oen I, Dokter J, Boxma H, van Beeck EF. Functional outcome after burns: a review. Burns. 2006;32(1):1-9.

For peer review only - http://bmjopen.bmj.com/site/about/guidelines.xhtml

18. Wasiak J, Cleland H, Campbell F, Spinks A. Dressings for superficial and partial thickness burns. The Cochrane Library. 2013.

19. Lawrence JW, Mason ST, Schomer K, Klein MB. Epidemiology and impact of scarring after burn injury: a systematic review of the literature. Journal of burn care & research. 2012;33(1):136-46.

20. Sinha IP, Smyth RL, Williamson PR. Using the Delphi technique to determine which outcomes to measure in clinical trials: recommendations for the future based on a systematic review of existing studies. PLoS medicine. 2011;8(1):e1000393.

21. Williamson PR, Altman DG, Blazeby JM, Clarke M, Devane D, Gargon E, et al. Developing core outcome sets for clinical trials: issues to consider. Trials. 2012;13(1):132.

22. Klein MB, Goverman J, Hayden DL, Fagan SP, McDonald-Smith GP, Alexander AK, et al. Benchmarking outcomes in the critically injured burn patient. Annals of surgery. 2014;259(5):833.

23. Pereira C, Murphy K, Herndon D. Outcome measures in burn care: is mortality dead? Burns. 2004;30(8):761-71.

24. Verbelen J, Hoeksema H, Heyneman A, Pirayesh A, Monstrey S. Aquacel® Ag dressing versus Acticoat™ dressing in partial thickness burns: a prospective, randomized, controlled study in 100 patients. Part 1: Burn wound healing. Burns. 2014;40(3):416-27.

25. Hop MJ, Polinder S, Vlies CH, Middelkoop E, Baar ME. Costs of burn care: a systematic review. Wound repair and regeneration. 2014;22(4):436-50.

26. Azzopardi EA, Azzopardi E, Camilleri L, Villapalos J, Boyce DE, Dziewulski P, et al. Gram negative wound infection in hospitalised adult burn patients-systematic review and metanalysis. PloS one. 2014;9(4):e95042.

27. Griffiths HR, Thornton K, Clements CM, Burge T, Kay A, Young A. The cost of a hot drink scald. Burns. 2006;32(3):372-4.

28. Holavanahalli RK, Helm PA, Kowalske KJ. Long-term outcomes in patients surviving large burns: the musculoskeletal system. Journal of Burn Care & Research. 2016;37(4):243-54.

29. Young A, Brookes S, Rumsey N, Blazeby J. Agreement on what to measure in randomised controlled trials in burn care: study protocol for the development of a core outcome set. BMJ open. 2017;7(6):e017267.

30. Hopkins J, Howes N, Chalmers K, Savovic J, Whale K, Coulman K, et al. Outcome reporting in bariatric surgery: an in‐depth analysis to inform the development of a core outcome set, the BARIACT S tudy. obesity reviews. 2015;16(1):88-106.

31. United Nations. [Available from: www.un.org/esa/population/publications/worldageing19502050/pdf/96annexii.pdf.

32. Abedini F, Ahmadi A, Yavari A, Hosseini V, Mousavi S. Comparison of silver nylon wound dressing and silver sulfadiazine in partial burn wound therapy. International wound journal. 2013;10(5):573-8.

33. Adhya A, Bain J, Ray O, Hazra A, Adhikari S, Dutta G, et al. Healing of burn wounds by topical treatment: A randomized controlled comparison between silver sulfadiazine and nano-crystalline silver. Journal of basic and clinical pharmacy. 2014;6(1):29.

34. Ahuja RB, Bansal P, Pradhan GS, Subberwal M. An analysis of deep vein thrombosis in burn patients (Part 1): Comparison of D-dimer and Doppler ultrasound as screening tools. Burns. 2016;42(8):1686-92.

For peer review only - http://bmjopen.bmj.com/site/about/guidelines.xhtml

35. Ahuja RB, Bansal P, Pradhan GS, Subberwal M. An analysis of deep vein thrombosis in burn patients (part II): A randomized and controlled study of thrombo-prophylaxis with low molecular weight heparin. Burns. 2016;42(8):1693-8.

36. Ahuja RB, Gupta GK. A four arm, double blind, randomized and placebo controlled study of pregabalin in the management of post-burn pruritus. Burns. 2013;39(1):24-9.

37. Ali A, Herndon DN, Mamachen A, Hasan S, Andersen CR, Grogans R-J, et al. Propranolol attenuates hemorrhage and accelerates wound healing in severely burned adults. Critical care. 2015;19(1):217.

38. Ali ZMI, El-Refay BH, Ali RR. Aerobic exercise training in modulation of aerobic physical fitness and balance of burned patients. Journal of physical therapy science. 2015;27(3):585-9.

39. Aramwit P, Palapinyo S, Srichana T, Chottanapund S, Muangman P. Silk sericin ameliorates wound healing and its clinical efficacy in burn wounds. Archives of dermatological research. 2013;305(7):585-94.

40. As'adi K, Emami SA, Salehi SH, Shoar S. A randomized controlled trial comparing endoscopic-assisted versus open neck tissue expander placement in reconstruction of post-burn facial scar deformities. Aesthetic plastic surgery. 2016;40(4):526-34.

41. Assadian O, Arnoldo B, Purdue G, Burris A, Skrinjar E, Duschek N, et al. A prospective, randomised study of a novel transforming methacrylate dressing compared with a silver‐containing sodium carboxymethylcellulose dressing on partial‐thickness skin graft donor sites in burn patients. International wound journal. 2015;12(3):351-6.

42. Bailey JK, Burkes SA, Visscher MO, Whitestone J, Kagan RJ, Yakuboff KP, et al. Multimodal Quantitative Analysis of Early Pulsed‐Dye Laser Treatment of Scars at a Pediatric Burn Hospital. Dermatologic Surgery. 2012;38(9):1490-6.

43. Béchir M, Puhan MA, Fasshauer M, Schuepbach RA, Stocker R, Neff TA. Early fluid resuscitation with hydroxyethyl starch 130/0.4 (6%) in severe burn injury: a randomized, controlled, double-blind clinical trial. Critical Care. 2013;17(6):R299.

44. Black JS, Drake DB. A prospective randomized trial comparing silver sulfadiazine cream with a water-soluble polyantimicrobial gel in partial-thickness burn wounds. Plastic Surgical Nursing. 2015;35(1):46-9.

45. Bloemen MC, Wal M, Verhaegen PD, Nieuwenhuis MK, Baar ME, Zuijlen PP, et al. Clinical effectiveness of dermal substitution in burns by topical negative pressure: a multicenter randomized controlled trial. Wound Repair and Regeneration. 2012;20(6):797-805.

46. Blome-Eberwein S, Abboud M, Lozano DD, Sharma R, Eid S, Gogal C. Effect of subcutaneous epinephrine/saline/local anesthetic versus saline-only injection on split-thickness skin graft donor site perfusion, healing, and pain. Journal of Burn Care & Research. 2013;34(2):e80-e6.

47. Brown MR, Dalziel SR, Herd E, Johnson K, Wong She R, Shepherd M. A randomized controlled study of silver-based burns dressing in a pediatric emergency department. Journal of Burn Care & Research. 2016;37(4):e340-e7.

48. Brown NJ, David M, Cuttle L, Kimble RM, Rodger S, Higashi H. Cost-effectiveness of a nonpharmacological intervention in pediatric burn care. Value in health. 2015;18(5):631-7.

49. Brown NJ, Kimble RM, Rodger S, Ware RS, Cuttle L. Play and heal: randomized controlled trial of Ditto™ intervention efficacy on improving re-epithelialization in pediatric burns. Burns. 2014;40(2):204-13.

50. Carr J, Crowley N. Prophylactic sequential bronchoscopy after inhalation injury: results from a three-year prospective randomized trial. European Journal of Trauma and Emergency Surgery. 2013;39(2):177-83.

For peer review only - http://bmjopen.bmj.com/site/about/guidelines.xhtml

For peer review only

51. Chahed J, Ksia A, Selmi W, Hidouri S, Sahnoun L, Krichene I, et al. Burns injury in children: is antibiotic prophylaxis recommended? African journal of paediatric surgery. 2014;11(4):323.

52. Chen G, Shao H, Pan X. A randomized controlled trial to compare the effects of liquid versus powdered recombinant human growth hormone in treating patients with severe burns. Biomedical reports. 2016;4(5):551-6.

53. Chen L, Meng K, Su W, Fu Y. The effect of continuous sedation therapy on immunomodulation, plasma levels of antioxidants, and indicators of tissue repair in post-burn sepsis patients. Cell biochemistry and biophysics. 2015;73(2):473-8.

54. Chen X, Feng X, Xie J, Ruan S, Lin Y, Lin Z, et al. Application of acellular dermal xenografts in full-thickness skin burns. Experimental and therapeutic medicine. 2013;6(1):194-8.

55. Chester SJ, Stockton K, De Young A, Kipping B, Tyack Z, Griffin B, et al. Effectiveness of medical hypnosis for pain reduction and faster wound healing in pediatric acute burn injury: study protocol for a randomized controlled trial. Trials. 2016;17(1):223.

56. Chi Y, Chai J, Luo H, Zhang Q, Feng R. Safety of recombinant human granulocyte-macrophage colony-stimulating factor in healing pediatric severe burns. Genet Mol Res. 2015;14(1):2735-41.

57. Cho YS, Choi YH. Comparison of three cooling methods for burn patients: A randomized clinical trial. Burns. 2017;43(3):502-8.

58. Cho YS, Jeon JH, Hong A, Yang HT, Yim H, Cho YS, et al. The effect of burn rehabilitation massage therapy on hypertrophic scar after burn: a randomized controlled trial. Burns. 2014;40(8):1513-20.

59. Cho YS, Joo SY, Cui H, Cho S-R, Yim H, Seo CH. Effect of extracorporeal shock wave therapy on scar pain in burn patients: a prospective, randomized, single-blind, placebo-controlled study. Medicine. 2016;95(32).

60. Chong SJ, Kan EM, Song C, Soh CR, Lu J. Characterization of early thermal burns and the effects of hyperbaric oxygen treatment: a pilot study. Diving and hyperbaric medicine. 2013:157.

61. Clayton RP, Wurzer P, Andersen CR, Mlcak RP, Herndon DN, Suman OE. Effects of different duration exercise programs in children with severe burns. Burns. 2017;43(4):796-803.

62. Cone L, Gottschlich M, Khoury J, Simakajornboon N, Kagan R. The Effect of Healing Touch on Sleep Patterns of Pediatric Burn Patients: A Prospective Pilot Study. J Sleep Disor: Treat Care 3: 2. of. 2014;6:2.

63. Csontos C, Rezman B, Foldi V, Bogar L, Drenkovics L, Röth E, et al. Effect of N-acetylcysteine treatment on oxidative stress and inflammation after severe burn. Burns. 2012;38(3):428-37.

64. Cucereanu-Bădică I, Luca I, Negreș S, Grințescu I, Lascăr I. The effects of intravenous glutamine supplementation in severely burned multiple traumatized patients. Farmacia; 2013.

65. Donovan ML, Muller MJ, Simpson C, Rudd M, Paratz J. Interim pressure garment therapy (4–6 mmHg) and its effect on donor site healing in burn patients: study protocol for a randomised controlled trial. Trials. 2016;17(1):214.

66. Ebid AA, Ahmed MT, Eid MM, Mohamed MSE. Effect of whole body vibration on leg muscle strength after healed burns: a randomized controlled trial. Burns. 2012;38(7):1019-26.

67. Ebid AA, El-Shamy SM, Draz AH. Effect of isokinetic training on muscle strength, size and gait after healed pediatric burn: A randomized controlled study. Burns. 2014;40(1):97-105.

For peer review only - http://bmjopen.bmj.com/site/about/guidelines.xhtml

68. Ebid AA, Omar MT, El Baky AMA. Effect of 12-week isokinetic training on muscle strength in adult with healed thermal burn. Burns. 2012;38(1):61-8.

69. Edionwe J, Hess C, Fernandez-Rio J, Herndon DN, Andersen CR, Klein GL, et al. Effects of whole-body vibration exercise on bone mineral content and density in thermally injured children. Burns. 2016;42(3):605-13.

70. Ehmer AIR, Mh; Khan, M; Hasan, F; Khaliq, Ra; Zehra, S. Efficacy of platelet rich plasma application in comparison to conventional dressing therapy in partial thickness burn wound. Medical Forum Monthly [Internet]. 2014; 25(1):[27-30 pp.]. Available from: http://onlinelibrary.wiley.com/o/cochrane/clcentral/articles/602/CN-01012602/frame.html.

71. El-Ghazely M, Mahmoud W, Atia M, Eldip E. Effect of probiotic administration in the therapy of pediatric thermal burn. Annals of burns and fire disasters. 2016;29(4):268.

72. Elsharnouby NM, Eid HE, Elezz NFA, Aboelatta YA. Heparin/N-acetylcysteine: an adjuvant in the management of burn inhalation injury: a study of different doses. Journal of critical care. 2014;29(1):182. e1-. e4.

73. Fatemi MJ, Salehi H, Akbari H, Alinejad F, Saberi M, Mousavi SJ, et al. The effect of levamisole on mortality rate among patients with severe burn injuries. Journal of research in medical sciences: the official journal of Isfahan University of Medical Sciences. 2013;18(9):795.

74. Finnerty CC, Ali A, McLean J, Benjamin N, Clayton RP, Andersen CR, et al. Impact of stress-induced diabetes on outcomes in severely burned children. Journal of the American College of Surgeons. 2014;218(4):783-95.

75. Gardien KL, Marck RE, Bloemen MC, Waaijman T, Gibbs S, Ulrich MM, et al. Outcome of burns treated with autologous cultured proliferating epidermal cells: a prospective randomized multicenter intrapatient comparative trial. Cell transplantation. 2016;25(3):437-48.

76. Kee EG, Kimble R, Cuttle L, Khan A, Stockton K. Randomized controlled trial of three burns dressings for partial thickness burns in children. Burns. 2015;41(5):946-55.

77. Genuino GAS, Baluyut-Angeles KV, Espiritu APT, Lapitan MCM, Buckley BS. Topical petrolatum gel alone versus topical silver sulfadiazine with standard gauze dressings for the treatment of superficial partial thickness burns in adults: a randomized controlled trial. Burns. 2014;40(7):1267-73.

78. Glas GJ, Muller J, Binnekade JM, Cleffken B, Colpaert K, Dixon B, et al. HEPBURN-investigating the efficacy and safety of nebulized heparin versus placebo in burn patients with inhalation trauma: study protocol for a multi-center randomized controlled trial. Trials. 2014;15(1):91.

79. Zhang Y, Gu F, Wang F, Zhang Y. Effects of early enteral nutrition on the gastrointestinal motility and intestinal mucosal barrier of patients with burn-induced invasive fungal infection. Pakistan journal of medical sciences. 2016;32(3):599.

80. Günter CI, Bader A, Dornseifer U, Egert S, Dunda S, Grieb G, et al. A multi-center study on the regenerative effects of erythropoietin in burn and scalding injuries: study protocol for a randomized controlled trial. Trials. 2013;14(1):124.

81. Hardee JP, Porter C, Sidossis LS, Børsheim E, Carson JA, Herndon DN, et al. Early rehabilitative exercise training in the recovery from pediatric burn. Medicine and science in sports and exercise. 2014;46(9):1710.

82. Hayashida K, Akita S. Quality of pediatric second-degree burn wound scars following the application of basic fibroblast growth factor: results of a randomized, controlled pilot study. Ostomy-Wound Management. 2012;58(8):32.

For peer review only - http://bmjopen.bmj.com/site/about/guidelines.xhtml

83. Herndon DN, Rodriguez NA, Diaz EC, Hegde S, Jennings K, Mlcak RP, et al. Long-term propranolol use in severely burned pediatric patients: a randomized controlled study. Annals of surgery. 2012;256(3):402.

84. Herndon DN, Voigt CD, Capek KD, Wurzer P, Guillory A, Kline A, et al. Reversal of growth arrest with the combined administration of oxandrolone and propranolol in severely burned children. Annals of surgery. 2016;264(3):421.

85. Hop MJ, Stekelenburg CM, Hiddingh J, Kuipers HC, Middelkoop E, Nieuwenhuis MK, et al. Cost-effectiveness of laser doppler imaging in burn care in the Netherlands: a randomized controlled trial. Plastic and reconstructive surgery. 2016;137(1):166e-76e.

86. Hsu K-C, Chen LF, Hsiep PH. Effect of music intervention on burn patients’ pain and anxiety during dressing changes. burns. 2016;42(8):1789-96.

87. Huang G, Liang B, Liu G, Liu K, Ding Z. Low dose of glucocorticoid decreases the incidence of complications in severely burned patients by attenuating systemic inflammation. Journal of critical care. 2015;30(2):436. e7-. e11.

88. Hyland EJ, D’Cruz R, Harvey JG, Moir J, Parkinson C, Holland AJ. An assessment of early Child Life Therapy pain and anxiety management: a prospective randomised controlled trial. Burns. 2015;41(8):1642-52.

89. Hyland EJ, D’Cruz R, Menon S, Chan Q, Harvey JG, Lawrence T, et al. Prospective, randomised controlled trial comparing Versajet™ hydrosurgery and conventional debridement of partial thickness paediatric burns. Burns. 2015;41(4):700-7.

90. Isaac K, Umraw N, Cartotto R. Construction of Skin Graft Seams in Burn Patients: A Prospective Randomized Double-Blinded Study. Journal of Burn Care & Research. 2016;37(6):397-403.

91. Jang KU, Choi JS, Mun JH, Jeon JH, Seo CH, Kim JH. Multi-axis shoulder abduction splint in acute burn rehabilitation: a randomized controlled pilot trial. Clinical rehabilitation. 2015;29(5):439-46.

92. Jeschke MG, Abdullahi A, Burnett M, Rehou S, Stanojcic M. Glucose control in severely burned patients using metformin: an interim safety and efficacy analysis of a phase II randomized controlled trial. Annals of surgery. 2016;264(3):518-27.

93. Jeschke MG, Williams FN, Finnerty CC, Rodriguez NA, Kulp GA, Ferrando A, et al. The effect of ketoconazole on post-burn inflammation, hypermetabolism and clinical outcomes. PloS one. 2012;7(5):e35465.

94. Kaheni S, Sadegh Rezai M, Bagheri-Nesami M, Goudarzian AH. The effect of Distraction Technique on the pain of Dressing change among 3-6 year-old children. International Journal of Pediatrics. 2016;4(4):1603-10.

95. Keorochana K, Chaiyasuk P, Muangman P. Prospective randomize-controlled comparison between silicone plus herbal extract gel versus Aloe vera gel for burn scar prophylaxis. Wound Medicine. 2015;9:1-4.

96. Kibor D, Nyaim O, Wanjeri K. Effects of Enteral Glutamine Supplementation on Reduction of Infection in Adult Patients with Severe Burns. East African medical journal. 2014;91(1):33-6.

97. Kim J-B, Cho YS, Jang KU, Joo SY, Choi JS, Seo CH. Effects of sustained release growth hormone treatment during the rehabilitation of adult severe burn survivors. Growth Hormone & IGF Research. 2016;27:1-6.

98. Kipping B, Rodger S, Miller K, Kimble RM. Virtual reality for acute pain reduction in adolescents undergoing burn wound care: a prospective randomized controlled trial. Burns. 2012;38(5):650-7.

99. Kolmus AM, Holland AE, Byrne MJ, Cleland HJ. The effects of splinting on shoulder function in adult burns. Burns. 2012;38(5):638-44.

For peer review only - http://bmjopen.bmj.com/site/about/guidelines.xhtml

100. Lagus H, Sarlomo-Rikala M, Böhling T, Vuola J. Prospective study on burns treated with Integra®, a cellulose sponge and split thickness skin graft: comparative clinical and histological study—randomized controlled trial. Burns. 2013;39(8):1577-87.

101. Lairet KF, Baer D, Leas ML, Renz EM, Cancio LC. Evaluation of an oxygen-diffusion dressing for accelerated healing of donor-site wounds. Journal of Burn Care & Research. 2014;35(3):214-8.

102. Lak S, Ostadrahimi A, Nagili B, Asghari-Jafarabadi M, Beigzali S, Salehi F, et al. Anti-Inflammatory effect of taurine in burned patients. Advanced pharmaceutical bulletin. 2015;5(4):531.

103. Lewis PA, Wright K, Webster A, Steer M, Rudd M, Doubrovsky A, et al. A randomized controlled pilot study comparing aqueous cream with a beeswax and herbal oil cream in the provision of relief from postburn pruritis. Journal of Burn Care & Research. 2012;33(4):e195-e200.

104. Li X, Meng X, Wang X, Li Y, Li W, Lv X, et al. Human acellular dermal matrix allograft: A randomized, controlled human trial for the long-term evaluation of patients with extensive burns. Burns. 2015;41(4):689-99.

105. López-Rodríguez L, Miguel A, García-Hierro P, Herrero R, Martins J, van Saene HK, et al. Selective digestive decontamination attenuates organ dysfunction in critically ill burn patients. Shock. 2016;46(5):492-7.

106. Lorello DJ, Peck M, Albrecht M, Richey KJ, Pressman MA. Results of a prospective randomized controlled trial of early ambulation for patients with lower extremity autografts. Journal of Burn Care & Research. 2014;35(5):431-6.

107. Mabrouk AB, Ns; Helal, Ha; Zaki, Bm; Maher, A. Moist occlusive dressing (Aquacel(®) Ag) versus moist open dressing (MEBO(®)) in the management of partial-thickness facial burns: a comparative study in Ain Shams University. Burns : journal of the International Society for Burn Injuries [Internet]. 2012; 38(3):[396-403 pp.]. Available from: http://onlinelibrary.wiley.com/o/cochrane/clcentral/articles/469/CN-00830469/frame.html.

108. Maghsoudi H, Nezami N, Mirzajanzadeh M. Enhancement of burn wounds healing by platelet dressing. International journal of burns and trauma. 2013;3(2):96.

109. Mahmoud W, Mostafa W, Abdel-Khalek A, Shalaby H. Effect of immune-enhancing diets on the outcomes of patients after major burns. Annals of burns and fire disasters. 2014;27(4):192.

110. Malin EW, Galin CM, Lairet KF, Huzar TF, Williams JF, Renz EM, et al. Silver-coated nylon dressing plus active DC microcurrent for healing of autogenous skin donor sites. Annals of plastic surgery. 2013;71(5):481-4.

111. Marck RE, Gardien KL, Stekelenburg CM, Vehmeijer M, Baas D, Tuinebreijer WE, et al. The application of platelet‐rich plasma in the treatment of deep dermal burns: A randomized, double‐blind, intra‐patient controlled study. Wound Repair and Regeneration. 2016;24(4):712-20.

112. Mayes T, Gottschlich MM, James LE, Allgeier C, Weitz J, Kagan RJ. Clinical safety and efficacy of probiotic administration following burn injury. Journal of Burn Care & Research. 2015;36(1):92-9.

113. McBride CA, Kimble RM, Stockton K. Three donor site dressings in pediatric split-thickness skin grafts: study protocol for a randomised controlled trial. Trials. 2015;16(1):43.

114. Moghazy A, Adly O, Elbadawy M, Hashem R. Evaluation of who oral rehydration solution (ORS) and salt tablets in resuscitating adult patients with burns covering more than 15% of total body surface area (TBSA). Annals of burns and fire disasters. 2016;29(1):43.

115. Mohamed SA-R, Mohamed NN. Efficacy and adverse events of early high-frequency oscillatory ventilation in adult burn patients with acute respiratory distress syndrome. Egyptian Journal of Anaesthesia. 2016;32(3):421-9.

For peer review only - http://bmjopen.bmj.com/site/about/guidelines.xhtml

116. Mohammadi AA, Johari HG, Eskandari S. Effect of amniotic membrane on graft take in extremity burns. Burns. 2013;39(6):1137-41.

117. Moravvej H, Hormozi AK, Hosseini SN, Sorouri R, Mozafari N, Ghazisaidi MR, et al. Comparison of the application of allogeneic fibroblast and autologous mesh grafting with the conventional method in the treatment of third-degree burns. Journal of Burn Care & Research. 2016;37(1):e90-e5.

118. Mowafy ZM, Monem MA, Zoheiry IMI, Khowailed KA-E, Ahmed BR. Effect of Low-Intensity Laser on the Neuropathic Common Peroneal Nerve Post Burn.

119. Muangman P, Praditsuktavorn B, Chinaroonchai K, Chuntrasakul C. Clinical efficacy test of polyester containing herbal extract dressings in burn wound healing. The international journal of lower extremity wounds. 2016;15(3):203-12.

120. Dittrich MHM, de Carvalho WB, Lavado EL. Evaluation of the "early" use of albumin in children with extensive burns: a randomized controlled trial. Pediatric critical care medicine. 2016;17(6):e280-e6.

121. Najmi M, Shariatpanahi ZV, Tolouei M, Amiri Z. Effect of oral olive oil on healing of 10–20% total body surface area burn wounds in hospitalized patients. Burns. 2015;41(3):493-6.

122. Nedelec B, Rachelska G, Parnell LK, LaSalle L. Double-blind, randomized, pilot study assessing the resolution of postburn pruritus. Journal of Burn Care & Research. 2012;33(3):398-406.

123. Nikkhah D, Booth S, Tay S, Gilbert P, Dheansa B. Comparing outcomes of sheet grafting with 1: 1 mesh grafting in patients with thermal burns: a randomized trial. Burns. 2015;41(2):257-64.

124. Noorbala MT, Noorbala M, Dashti-Rahmatabadi MH, Noorbala M, Noorbala R, Mozaffary B. Comparison of Hydrogel Produced by Radiation as Applied at the Research Center (Yazd Branch) With MaxGel and Routine Dressing for Second-Degree Burn Repair in Yazd Burn Hospital. Iranian Red Crescent Medical Journal. 2016;18(8).

125. Oen IM, van Baar ME, Middelkoop E, Nieuwenhuis MK. Effectiveness of Cerium Nitrate–Silver Sulfadiazine in the Treatment of Facial Burns: A Multicenter, Randomized, Controlled Trial. Plastic and reconstructive surgery. 2012;130(2):274e-83e.

126. Omar MT, Hegazy FA, Mokashi SP. Influences of purposeful activity versus rote exercise on improving pain and hand function in pediatric burn. Burns. 2012;38(2):261-8.

127. Orrey DC, Halawa OI, Bortsov AV, Shupp JW, Jones SW, Haith LR, et al. Results of a pilot multi-center genotype-based randomized placebo-controlled trial of propranolol to reduce pain after major thermal burn injury. The Clinical journal of pain. 2015;31(1):21.

128. Omranifard M, Heidari M, Farajzadegan Z, Niktabar MR, Motamedi N. Botulinum Toxin and Burn Induces Contracture. Archives of plastic surgery. 2016;43(6):609-11.

129. Ostadrahimi A, Nagili B, Asghari-Jafarabadi M, Beigzali S, Zalouli H, Lak S. A Proper Enteral Nutrition Support Improves Sequential Organ Failure Score and Decreases Length of Stay in Hospital in Burned Patients. Iranian Red Crescent Medical Journal. 2016;18(2).

130. Ostlie DJ, Juang D, Aguayo P, Pettiford-Cunningham JP, Erkmann EA, Rash DE, et al. Topical silver sulfadiazine vs collagenase ointment for the treatment of partial thickness burns in children: a prospective randomized trial. Journal of pediatric surgery. 2012;47(6):1204-7.

131. Ottomann C, Stojadinovic A, Lavin PT, Gannon FH, Heggeness MH, Thiele R, et al. Prospective randomized phase II trial of accelerated reepithelialization of superficial second-degree burn wounds using extracorporeal shock wave therapy. Annals of surgery. 2012;255(1):23-9.

For peer review only

132. Ouyang J, Chen Y-c, Luo G-x, Yan H, Peng Y-z, Huang Y-s, et al. A randomized and controlled multicenter prospective study of the chinese medicinal compound fufang xuelian burn ointment for the treatment of superficial and deep second-degree burn wounds. Cell biochemistry and biophysics. 2014;69(3):467-74.

133. Palmieri TL, Greenhalgh DG, Sen S. Prospective comparison of packed red blood cell–to–fresh frozen plasma transfusion ratio of 4: 1 versus 1: 1 during acute massive burn excision. Journal of Trauma and Acute Care Surgery. 2013;74(1):76-83.

134. Panahi Y, Beiraghdar F, Akbari H, Bekhradi H, Taghizadeh M, Sahebkar A. A herbal cream consisting of Aloe vera, Lavandula stoechas, and Pelargonium roseum as an alternative for silver sulfadiazine in burn management. Asian Biomedicine. 2012;6(2):273-8.

135. Parker M, Delahunty B, Heberlein N, Devenish N, Wood FM, Jackson T, et al. Interactive gaming consoles reduced pain during acute minor burn rehabilitation: a randomized, pilot trial. Burns. 2016;42(1):91-6.

136. Parry I, Painting L, Bagley A, Kawada J, Molitor F, Sen S, et al. A pilot prospective randomized control trial comparing exercises using videogame therapy to standard physical therapy: 6 months follow-up. Journal of Burn Care & Research. 2015;36(5):534-44.

137. Patton ML, Mullins RF, Smith D, Korentager R. An open, prospective, randomized pilot investigation evaluating pain with the use of a soft silicone wound contact layer vs bridal veil and staples on split thickness skin grafts as a primary dressing. Journal of Burn Care & Research. 2013;34(6):674-81.

138. Peña R, Ramirez LL, Crandall CG, Wolf SE, Herndon DN, Suman OE. Effects of community-based exercise in children with severe burns: a randomized trial. Burns. 2016;42(1):41-7.

139. Porro LJ, Al-Mousawi AM, Williams F, Herndon DN, Mlcak RP, Suman OE. Effects of propranolol and exercise training in children with severe burns. The Journal of pediatrics. 2013;162(4):799-803. e1.

140. Porro LJ, Herndon DN, Rodriguez NA, Jennings K, Klein GL, Mlcak RP, et al. Five-year outcomes after oxandrolone administration in severely burned children: a randomized clinical trial of safety and efficacy. Journal of the American College of Surgeons. 2012;214(4):489-502.

141. Santos Portilla A, Bravo GL, Miraval FK, Villamar MF, Schneider JC, Ryan CM, et al. A feasibility study assessing cortical plasticity in chronic neuropathic pain following burn injury. Journal of Burn Care & Research. 2013;34(1):e48-e52.

142. Rashaan ZM, Krijnen P, van den Akker-van ME, van Baar ME, Vloemans AF, Dokter J, et al. Clinical effectiveness, quality of life and cost-effectiveness of Flaminal® versus Flamazine® in the treatment of partial thickness burns: study protocol for a randomized controlled trial. Trials. 2016;17(1):122.

143. Reeves PT, Herndon DN, Tanksley JD, Jennings K, Klein GL, Mlcak RP, et al. Five-year outcomes after long-term oxandrolone administration in severely burned children: a randomized clinical trial. Shock (Augusta, Ga). 2016;45(4):367.

144. Rosenberg L, Krieger Y, Bogdanov-Berezovski A, Silberstein E, Shoham Y, Singer AJ. A novel rapid and selective enzymatic debridement agent for burn wound management: a multi-center RCT. Burns. 2014;40(3):466-74.

145. Rousseau A-F, Foidart-Desalle M, Ledoux D, Remy C, Croisier J-L, Damas P, et al. Effects of cholecalciferol supplementation and optimized calcium intakes on vitamin D status, muscle strength and bone health: a one-year pilot randomized controlled trial in adults with severe burns. Burns. 2015;41(2):317-25.

146. Salehi SH, As'adi K, Mousavi SJ, Shoar S. Evaluation of amniotic membrane effectiveness in skin graft donor site dressing in burn patients. Indian Journal of Surgery. 2015;77(2):427-31.

For peer review only

147. Schaden E, Kimberger O, Kraincuk P, Baron D, Metnitz P, Kozek-Langenecker S. Perioperative treatment algorithm for bleeding burn patients reduces allogeneic blood product requirements. British journal of anaesthesia. 2012;109(3):376-81.

148. Schulz A, Depner C, Lefering R, Kricheldorff J, Kästner S, Fuchs PC, et al. A prospective clinical trial comparing Biobrane® Dressilk® and PolyMem® dressings on partial-thickness skin graft donor sites. Burns. 2016;42(2):345-55.

149. Shahzad MN, Ahmed N. Effectiveness of Aloe vera gel compared with 1% silver sulphadiazine cream as burn wound dressing in second degree burns. J Pak Med Assoc. 2013;63(2):225-30.

150. Siadat AH, Iraji F, Bahrami R, Nilfroushzadeh MA, Asilian A, Shariat S, et al. The comparison between modified kligman formulation versus kligman formulation and intense pulsed light in the treatment of the post-burn hyperpigmentation. Advanced biomedical research. 2016;5.

151. Dallal MS, Safdari R, Koochak HE, Sharifi-Yazdi S, Akhoondinasab M, Pourmand M, et al. A comparison between occlusive and exposure dressing in the management of burn wound. Burns. 2016;42(3):578-82.

152. Sood R, Roggy DE, Zieger MJ, Nazim M, Hartman BC, Gibbs JT. A comparative study of spray keratinocytes and autologous meshed split-thickness skin graft in the treatment of acute burn injuries. Wounds: a compendium of clinical research and practice. 2015;27(2):31-40.

153. Sousse LE, Herndon DN, Mlcak RP, Lee JO, Andersen CR, Zovath AJ, et al. Long-term administration of oxandrolone improves lung function in pediatric burned patients. Journal of Burn Care & Research. 2016;37(5):273-7.

154. Tang H, Lv G, Fu J, Niu X, Li Y, Zhang M, et al. An open, parallel, randomized, comparative, multicenter investigation evaluating the efficacy and tolerability of Mepilex Ag versus silver sulfadiazine in the treatment of deep partial-thickness burn injuries. Journal of Trauma and Acute Care Surgery. 2015;78(5):1000-7.

155. Taudorf EH, Danielsen PL, Paulsen IF, Togsverd‐Bo K, Dierickx C, Paasch U, et al. Non‐ablative fractional laser provides long‐term improvement of mature burn scars‐A randomized controlled trial with histological assessment. Lasers in surgery and medicine. 2015;47(2):141-7.

156. Tokarik M, Sjöberg F, Balik M, Pafcuga I, Broz L. Fluid therapy LiDCO controlled trial—optimization of volume resuscitation of extensively burned patients through noninvasive continuous real-time hemodynamic monitoring LiDCO. Journal of Burn Care & Research. 2013;34(5):537-42.

157. Vejdan SA, Khosravi M, Zojaji F. Burn Donor Site Dressing Using Melolin and Flexigrid Versus Conventional Dressing. Shiraz E-Medical Journal. 2015;16(1).

158. Venclauskiene A, Basevicius A, Zacharevskij E, Vaicekauskas V, Rimdeika R, Lukosevicius S. Laser Doppler imaging as a tool in the burn wound treatment protocol. Videosurgery and Other Miniinvasive Techniques. 2014;9(1):24.

159. Venet F, Plassais J, Textoris J, Cazalis M-A, Pachot A, Bertin-Maghit M, et al. Low-dose hydrocortisone reduces norepinephrine duration in severe burn patients: a randomized clinical trial. Critical Care. 2015;19(1):21.

160. Venkatachalapathy T. A comparative study of paediatric thermal burns treated with topical heparin and without heparin. Indian Journal of Surgery. 2014;76(4):282-7.

161. Verhaegen PD, Bloemen MC, van der Wal MB, Vloemans AF, Tempelman FR, Beerthuizen GI, et al. Skin stretching for primary closure of acute burn wounds. Burns. 2014;40(8):1727-37.

162. Voon K, Silberstein I, Eranki A, Phillips M, Wood FM, Edgar DW. Xbox Kinect™ based rehabilitation as a feasible adjunct for minor upper limb burns rehabilitation: A pilot RCT. Burns. 2016;42(8):1797-804.

For peer review only - http://bmjopen.bmj.com/site/about/guidelines.xhtml

163. Wang C, Wang J, Feng J. Local application of low-dose insulin in improving wound healing after deep burn surgery. Experimental and therapeutic medicine. 2016;12(4):2527-30.

164. Wibbenmeyer L, Eid A, Liao J, Heard J, Horsfield A, Kral L, et al. Gabapentin is ineffective as an analgesic adjunct in the immediate postburn period. Journal of Burn Care & Research. 2014;35(2):136-42.

165. Wood F, Martin L, Lewis D, Rawlins J, McWilliams T, Burrows S, et al. A prospective randomised clinical pilot study to compare the effectiveness of Biobrane® synthetic wound dressing, with or without autologous cell suspension, to the local standard treatment regimen in paediatric scald injuries. Burns. 2012;38(6):830-9.

166. Wu J, Xu R, Zhan R, Luo G, Niu X, Liu Y, et al. Effective symptomatic treatment for severe and intractable pruritus associated with severe burn-induced hypertrophic scars: A prospective, multicenter, controlled trial. Burns. 2016;42(5):1059-66.

167. Wurzer P, Branski LK, Clayton RP, Hundeshagen G, Forbes AA, Voigt CD, et al. Propranolol reduces cardiac index but does not adversely affect peripheral perfusion in severely burned children. Shock (Augusta, Ga). 2016;46(5):486.

168. Xiao H, Li C, Zhou X, Wang X, Wu Z, Zhang L, et al. A new method of microskin autografting with a Vaseline-based moisture dressing on granulation tissue. Burns. 2014;40(2):337-46.

169. Yan H, Chen J, Peng X. Recombinant human granulocyte-macrophage colony-stimulating factor hydrogel promotes healing of deep partial thickness burn wounds. Burns. 2012;38(6):877-81.

170. Yarboro DD. A comparative study of the dressings silver sulfadiazine and Aquacel Ag in the management of superficial partial-thickness burns. Advances in skin & wound care. 2013;26(6):259-62.

171. Yim H, Yang H-T, Cho Y-S, Kim D, Kim J-H, Chun W, et al. A clinical trial designed to evaluate the safety and effectiveness of a thermosensitive hydrogel-type cultured epidermal allograft for deep second-degree burns. Burns. 2014;40(8):1642-9.

172. Yuan L, Minghua C, Feifei D, Runxiu W, Ziqiang L, Chengyue M, et al. Study of the use of recombinant human granulocyte-macrophage colony-stimulating factor hydrogel externally to treat residual wounds of extensive deep partial-thickness burn. Burns. 2015;41(5):1086-91.

173. Zahmatkesh M, Manesh MJ, Babashahabi R. Effect of Olea ointment and Acetate Mafenide on burn wounds–A randomized clinical trial. Iranian journal of nursing and midwifery research. 2015;20(5):599.

174. Zhang J, Li X, Gao Y, Guo G, Xu C, Li G, et al. Effects of puerarin on the inflammatory role of burn-related procedural pain mediated by P2X7 receptors. Burns. 2013;39(4):610-8.

175. Zidan SM, Eleowa SA, Nasef MA, Abd-Almoktader MA, Elbatawy AM, Borhamy AG, et al. Maximizing the safety of glycerol preserved human amniotic membrane as a biological dressing. Burns. 2015;41(7):1498-503.

176. Zu H, Li Q, Huang P, Wang X. Therapeutic value of blood purification and prognostic utilities of early serum procalcitonin, C reactive protein, and brain natriuretic peptide levels in severely burned patients with sepsis. Cell biochemistry and biophysics. 2015;72(1):259-63.

177. Wasiak J, Cleland H, Campbell F. Dressings for superficial and partial thickness burns. Cochrane Database Syst Rev. 2008;4.

178. Clare G, Suleman H, Bunce C, Dua H. Amniotic membrane transplantation for acute ocular burns. Cochrane Database of Systematic Reviews. 2011;9.

179. Breederveld RS, Tuinebreijer WE. Recombinant human growth hormone for treating burns and donor sites. The Cochrane Library. 2014.

For peer review only - http://bmjopen.bmj.com/site/about/guidelines.xhtml

180. Dumville JC, Munson C. Negative pressure wound therapy for partial-thickness burns. Cochrane Database Syst Rev. 2012;12.

181. Barajas-Nava LA, Lopez-Alcalde J, Roqué i Figuls M, Solà I, Bonfill Cosp X. Antibiotic prophylaxis for preventing burn wound infection. Cochrane Database of Systematic Reviews. 2013;6.

182. Hoogewerf CJ, Van Baar ME, Hop MJ, Nieuwenhuis MK, Oen IM, Middelkoop E. Topical treatment for facial burns. The Cochrane Library. 2013.

183. Wasiak J, Mahar PD, McGuinness SK, Spinks A, Danilla S, Cleland H, et al. Intravenous lidocaine for the treatment of background or procedural burn pain. The Cochrane Library. 2014.

184. Tan HB, Danilla S, Murray A, Serra R, El Dib R, Henderson TO, et al. Immunonutrition as an adjuvant therapy for burns. The Cochrane Library. 2014.

185. Masters B, Wood F, Tuckerman J, Peng L. High carbohydrate, high protein, low fat versus low carbohydrate, high protein, high fat enteral feeds for burns. status and date: Edited (no change to conclusions), published in. 2008(3).

186. Potter S, Brigic A, Whiting P, Cawthorn S, Avery K, Donovan J, et al. Reporting clinical outcomes of breast reconstruction: a systematic review. Journal of the National Cancer Institute. 2010;103(1):31-46.

187. Blencowe NS, Strong S, McNair AG, Brookes ST, Crosby T, Griffin SM, et al. Reporting of short-term clinical outcomes after esophagectomy: a systematic review. Ann Surg. 2012;255(4):658-66.

188. Whitehead L, Perkins G, Clarey A, Haywood K. A systematic review of the outcomes reported in cardiac arrest clinical trials: the need for a core outcome set. Resuscitation. 2015;88:150-7.

189. Hayes J, Black N, Jenkinson C, Young J, Rowan K, Daly K, et al. Outcome measures for adult critical care: a systematic review. Health technology assessment (Winchester, England). 2000;4(24):1-111.

190. Zakrzewska JM, Lopez BC. Quality of reporting in evaluations of surgical treatment of trigeminal neuralgia: recommendations for future reports. Neurosurgery. 2003;53(1):110-22.

191. Riddle DL, Stratford PW, Bowman DH. Findings of extensive variation in the types of outcome measures used in hip and knee replacement clinical trials: a systematic review. Arthritis care & research. 2008;59(6):876-83.

192. McNaughton-Collins M, Walker-Corkery E, Barry MJ. Health-related quality of life, satisfaction, and economic outcome measures in studies of prostate cancer screening and treatment, 1990-2000. JNCI Monographs. 2004;2004(33):78-101.

193. Deyo RA, Battie M, Beurskens A, Bombardier C, Croft P, Koes B, et al. Outcome measures for low back pain research: a proposal for standardized use. Spine. 1998;23(18):2003-13.

194. Bruce J, Russell E, Mollison J, Krukowski Z. The quality of measurement of surgical wound infection as the basis for monitoring: a systematic review. Journal of Hospital Infection. 2001;49(2):99-108.

195. Bruce J, Krukowski ZH, Al‐Khairy G, Russell EM, Park K. Systematic review of the definition and measurement of anastomotic leak after gastrointestinal surgery. British Journal of Surgery. 2001;88(9):1157-68.

196. Dodd S, Clarke M, Becker L, Mavergames C, Fish R, Williamson PR. A taxonomy has been developed for outcomes in medical research to help improve knowledge discovery. Journal of clinical epidemiology. 2017.

197. Clarke M. Standardising outcomes for clinical trials and systematic reviews. Trials. 2007;8(1):39.

For peer review only - http://bmjopen.bmj.com/site/about/guidelines.xhtml

198. Clarke M. Standardising outcomes in paediatric clinical trials. PLoS Medicine. 2008;5(4):e102.

199. Kirkham JJ, Boers M, Tugwell P, Clarke M, Williamson PR. Outcome measures in rheumatoid arthritis randomised trials over the last 50 years. Trials. 2013;14(1):324.

200. Bautista-Molano W, Navarro-Compán V, Landewé RB, Boers M, Kirkham JJ, van der Heijde D. How well are the ASAS/OMERACT Core Outcome Sets for Ankylosing Spondylitis implemented in randomized clinical trials? A systematic literature review. Clinical rheumatology. 2014;33(9):1313-22.

201. Falder S, Browne A, Edgar D, Staples E, Fong J, Rea S, et al. Core outcomes for adult burn survivors: a clinical overview. Burns. 2009;35(5):618-41.

202. Kirkham JJ, Davis K, Altman DG, Blazeby JM, Clarke M, Tunis S, et al. Core Outcome Set-STAndards for Development: The COS-STAD recommendations. PLoS medicine. 2017;14(11):e1002447.

203. Kirkham JJ, Gorst S, Altman DG, Blazeby JM, Clarke M, Devane D, et al. Core outcome set–STAndards for reporting: the COS-STAR statement. PLoS medicine. 2016;13(10):e1002148.

204. (COMET) COMiET.

205. Gargon E, Williamson PR, Altman DG, Blazeby JM, Clarke M. The COMET Initiative database: progress and activities from 2011 to 2013. Trials. 2014;15(1):279.

206. Chiarotto A, Ostelo RW, Turk DC, Buchbinder R, Boers M. Core outcome sets for research and clinical practice. Brazilian journal of physical therapy. 2017;21(2):77-84.

207. Gorst SL, Gargon E, Clarke M, Blazeby JM, Altman DG, Williamson PR. Choosing important health outcomes for comparative effectiveness research: an updated review and user survey. PLoS One. 2016;11(1):e0146444.

208. Biggane AM, Brading L, Ravaud P, Young B, Williamson PR. Survey indicated that core outcome set development is increasingly including patients, being conducted internationally and using Delphi surveys. Trials. 2018;19(1):113.

For peer review only

For peer review only - http://bmjopen.bmj.com/site/about/guidelines.xhtml

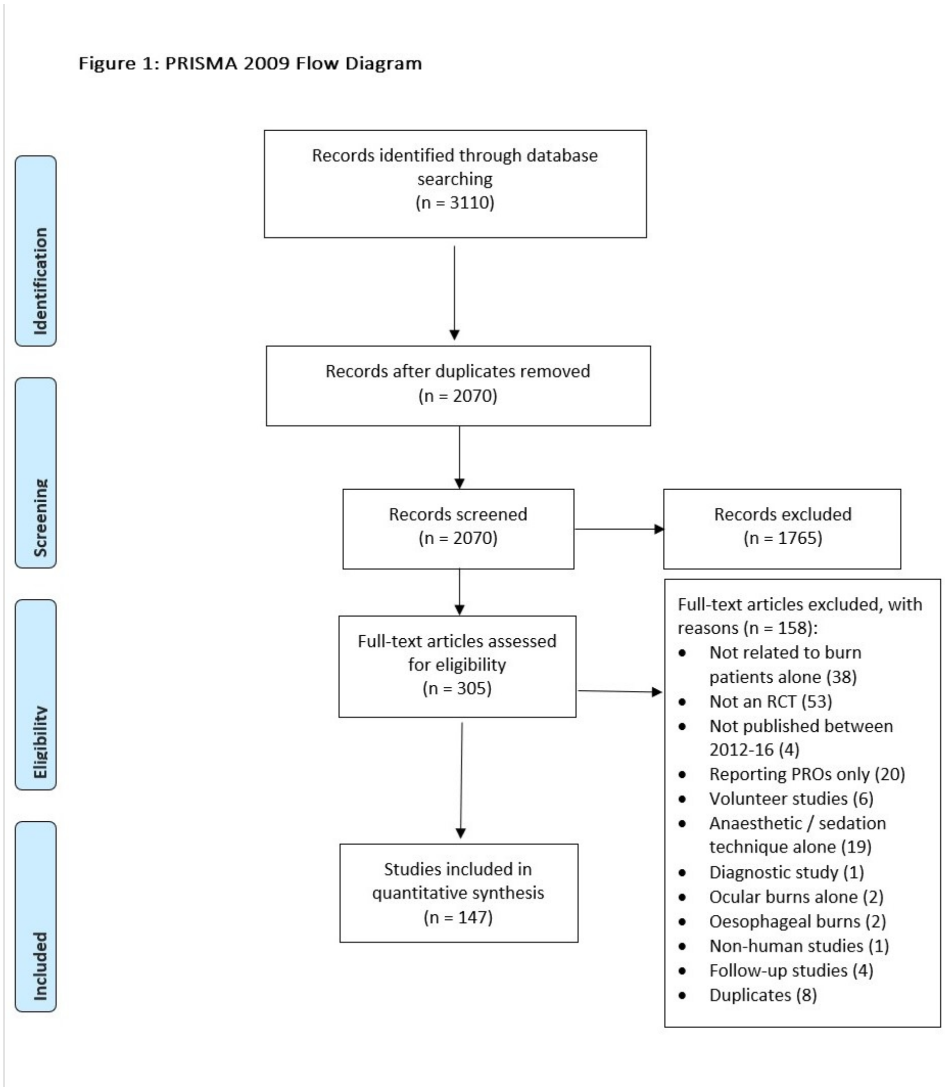

Figure 1: PRISMA 2009 Flow Diagram

259x280mm (96 x 96 DPI)

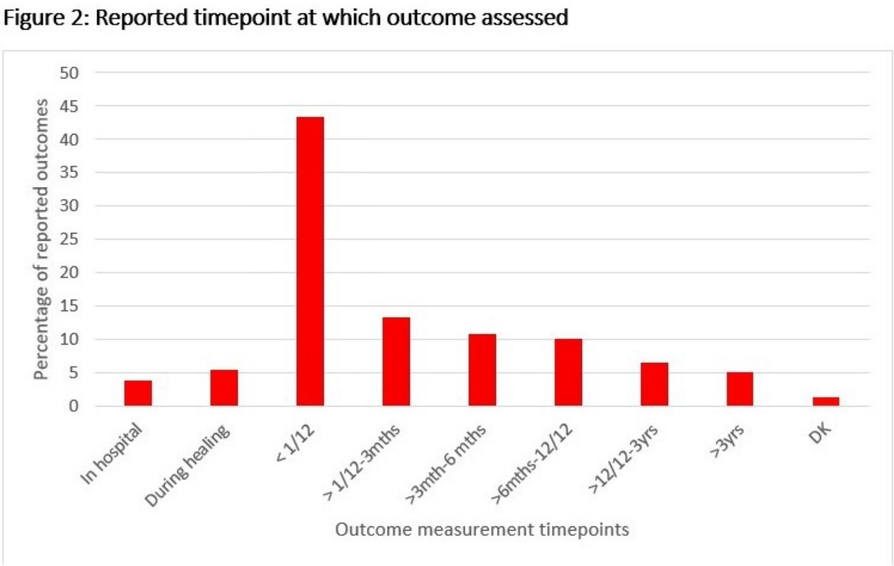

Figure 2: reported timepoint at which outcome assessed.

221x132mm (96 x 96 DPI)

Appendix A

Search Strategy using Ovid MEDLINE:

1. exp Burns/
2. burn*.tw.
3. scald*.tw.
4. (thermal* adj injur*).mp. [mp=title, abstract, original title, name of substance word, subject heading word, keyword heading word, protocol supplementary concept word, rare disease supplementary concept word, unique identifier, synonyms]
5. (smoke adj inhalation).mp. [mp=title, abstract, original title, name of substance word, subject heading word, keyword heading word, protocol supplementary concept word, rare disease supplementary concept word, unique identifier, synonyms]
6. 1 or 2 or 3 or 4 or 5
7. heartburn.mp. [mp=title, abstract, original title, name of substance word, subject heading word, keyword heading word, protocol supplementary concept word, rare disease supplementary concept word, unique identifier, synonyms]
8. burnout.mp. [mp=title, abstract, original title, name of substance word, subject heading word, keyword heading word, protocol supplementary concept word, rare disease supplementary concept word, unique identifier, synonyms]
9. (burn* adj out).mp. [mp=title, abstract, original title, name of substance word, subject heading word, keyword heading word, protocol supplementary concept word, rare disease supplementary concept word, unique identifier, synonyms]
10. burning.mp. [mp=title, abstract, original title, name of substance word, subject heading word, keyword heading word, protocol supplementary concept word, rare disease supplementary concept word, unique identifier, synonyms]
11. burnetii.mp. [mp=title, abstract, original title, name of substance word, subject heading word, keyword heading word, protocol supplementary concept word, rare disease supplementary concept word, unique identifier, synonyms]
12. burnish*.mp. [mp=title, abstract, original title, name of substance word, subject heading word, keyword heading word, protocol supplementary concept word, rare disease supplementary concept word, unique identifier, synonyms]
13. burnet*.mp. [mp=title, abstract, original title, name of substance word, subject heading word, keyword heading word, protocol supplementary concept word, rare disease supplementary concept word, unique identifier, synonyms]
14. 7 or 8 or 9 or 10 or 11 or 12 or 13
15. 6 not 14
16. (randomi?ed adj control* adj trial*).mp. [mp=title, abstract, original title, name of substance word, subject heading word, keyword heading word, protocol supplementary concept word, rare disease supplementary concept word, unique identifier, synonyms]
17. RCT.tw.
18. trial.tw.
19. 16 or 17 or 18
20. 15 and 19
21. limit 20 to (english language and humans and yr="2012 - 2016")
22. limit 21 to randomized controlled trial

For peer review only

PRISMA-P (Preferred Reporting Items for Systematic review and Meta-Analysis Protocols) 2015 checklist: recommended items to address in a systematic review protocol*

Section and topic	Item No	Checklist item	
**ADMINISTRATIVE INFORMATION**			
Title:			
Identification	1a	Identify the report as a protocol of a systematic review	p1
Update	1b	If the protocol is for an update of a previous systematic review, identify as such	N/A
Registration	2	If registered, provide the name of the registry (such as PROSPERO) and registration number	p10
Authors:			
Contact	3a	Provide name, institutional affiliation, e-mail address of all protocol authors; provide physical mailing address of corresponding author	p1
Contributions	3b	Describe contributions of protocol authors and identify the guarantor of the review	p10
Amendments	4	If the protocol represents an amendment of a previously completed or published protocol, identify as such and list changes; otherwise, state plan for documenting important protocol amendments	
Support:			
Sources	5a	Indicate sources of financial or other support for the review	p10
Sponsor	5b	Provide name for the review funder and/or sponsor	p10
Role of sponsor or funder	5c	Describe roles of funder(s), sponsor(s), and/or institution(s), if any, in developing the protocol	
**INTRODUCTION**			
Rationale	6	Describe the rationale for the review in the context of what is already known	p3
Objectives	7	Provide an explicit statement of the question(s) the review will address with reference to participants, interventions, comparators, and outcomes (PICO)	p4
**METHODS**			
Eligibility criteria	8	Specify the study characteristics (such as PICO, study design, setting, time frame) and report characteristics (such as years considered, language, publication status) to be used as criteria for eligibility for the review	p4
Information sources	9	Describe all intended information sources (such as electronic databases, contact with study authors, trial registers or other grey literature sources) with planned dates of coverage	p4
Search strategy	10	Present draft of search strategy to be used for at least one electronic database, including planned limits, such that it could be repeated	Appendix A
Study records:			
Data management	11a	Describe the mechanism(s) that will be used to manage records and data throughout the review	p5

For peer review only

Selection process	11b	State the process that will be used for selecting studies (such as two independent reviewers) through each phase of the review (that is, screening, eligibility and inclusion in meta-analysis)	p5
Data collection process	11c	Describe planned method of extracting data from reports (such as piloting forms, done independently, in duplicate), any processes for obtaining and confirming data from investigators	p5
Data items	12	List and define all variables for which data will be sought (such as PICO items, funding sources), any pre-planned data assumptions and simplifications	p5
Outcomes and prioritization	13	List and define all outcomes for which data will be sought, including prioritization of main and additional outcomes, with rationale	p5-6
Risk of bias in individual studies	14	Describe anticipated methods for assessing risk of bias of individual studies, including whether this will be done at the outcome or study level, or both; state how this information will be used in data synthesis	p5
Data synthesis	15a	Describe criteria under which study data will be quantitatively synthesised	N/A
	15b	If data are appropriate for quantitative synthesis, describe planned summary measures, methods of handling data and methods of combining data from studies, including any planned exploration of consistency (such as I^2, Kendall's τ)	
	15c	Describe any proposed additional analyses (such as sensitivity or subgroup analyses, meta-regression)	
	15d	If quantitative synthesis is not appropriate, describe the type of summary planned	
Meta-bias(es)	16	Specify any planned assessment of meta-bias(es) (such as publication bias across studies, selective reporting within studies)	
Confidence in cumulative evidence	17	Describe how the strength of the body of evidence will be assessed (such as GRADE)	

*** It is strongly recommended that this checklist be read in conjunction with the PRISMA-P Explanation and Elaboration (cite when available) for important clarification on the items. Amendments to a review protocol should be tracked and dated. The copyright for PRISMA-P (including checklist) is held by the PRISMA-P Group and is distributed under a Creative Commons Attribution Licence 4.0.**

From: Shamseer L, Moher D, Clarke M, Ghersi D, Liberati A, Petticrew M, Shekelle P, Stewart L, PRISMA-P Group. Preferred reporting items for systematic review and meta-analysis protocols (PRISMA-P) 2015: elaboration and explanation. BMJ. 2015 Jan 2;349(jan02 1):g7647.

For peer review only

A systematic review of clinical outcome reporting in randomised controlled trials of burn care.

Journal:	*BMJ Open*
Manuscript ID	bmjopen-2018-025135.R1
Article Type:	Research
Date Submitted by the Author:	20-Nov-2018
Complete List of Authors:	Young, Amber; University Hospitals Bristol NHS Foundation Trust, Paediatric Anaesthesia; University of Bristol, Canynge Hall<br>Davies, Anna; University of Bristol, Population Health Sciences<br>Bland, Sophie; West of England Academic Health Science Network<br>Brookes, Sara; Institute of Cancer and Genomic Sciences, University of Birmingham, Cancer Research UK Clinical Trials Unit ; University of Bristol, Population Health Sciences<br>Blazeby, Jane; University of Bristol, Population Health Sciences
**Primary Subject Heading**:	Research methods
Secondary Subject Heading:	Evidence based practice, Research methods, Surgery
Keywords:	Clinical decision-making, Review, Treatment Outcome, Outcome Assessment (Health Care), Burns, Randomised Controlled Trials as Topic

SCHOLARONE™
Manuscripts

A systematic review of clinical outcome reporting in randomised controlled trials of burn care.

Amber E Young[1], Anna Davies[1], Sophie Bland[2], Sara Brookes[1,3], Jane M Blazeby[1]

[1]Population Health Sciences, Bristol Medical School, University of Bristol, Canynge Hall, Bristol, UK

[2]Patient representative

[3]Cancer Research UK Clinical Trials Unit (CRCTU), Institute of Cancer and Genomic Sciences, University of Birmingham, Edgbaston, Birmingham, UK

Amber.young1@nhs.net

Anna.davies@bristol.ac.uk

Sophie.bland@weahsn.net

s.t.brookes@bham.ac.uk

j.m.blazeby@bristol.ac.uk

Corresponding author:

Dr Amber Elizabeth Young

Population Health Sciences, Bristol Medical School, University of Bristol, Canynge Hall, Bristol, UK

amber.young1@nhs.net

0117 3427900

For peer review only

Abstract

Systematic reviews collate trial data to provide evidence to support clinical decision-making. For effective synthesis, there must be consistency in outcome reporting. There is no agreed set of outcomes for reporting the effect of burn care interventions. Issues with outcome reporting have been identified, although not systematically investigated. This study gathers empirical evidence on any variation in outcome reporting and assesses the need for a Core Outcome Set for burn care research.

Methods: Electronic searches of four search engines were undertaken from January 2012 to December 2016 for RCTs, using medical subject headings and free text terms including ‘burn’, ‘scald’ ‘thermal injury’ and ‘RCT’. Two authors independently screened papers, extracted outcomes verbatim and recorded the timing of outcome measurement. Duplicate outcomes (exact wording +/- different spelling), similar outcomes (albumin in blood, serum albumin) and identical outcomes measured at different times were removed. Variation in outcome

For peer review only - http://bmjopen.bmj.com/site/about/guidelines.xhtml

reporting was determined by assessing the number of unique outcomes reported across all included trials. Outcomes were classified into domains. Bias was reduced using five researchers and a patient working independently and together.

Results: 147 trials were included, of which 127 (86.4%) were RCTs, 13 (8.8%) pilot studies and 7 (4.8%) RCT protocols. 1,494 verbatim clinical outcomes were reported; 955 were unique. 76.8% of outcomes were measured within six months of injury. Commonly reported outcomes were defined differently. Numbers of unique outcomes per trial varied from one to 37 (median 9; IQR 8). No single outcome was reported across all studies demonstrating inconsistency of reporting. Outcomes were classified into 54 domains. Numbers of outcomes per domain ranged from 1 to 166 (median 11; IQR 21).

Conclusions: This review has demonstrated heterogeneity in outcome reporting in burn care research which will hinder amalgamation of study data. We recommend the development of a Core Outcome Set.

Key words: Clinical decision-making, Review, Treatment Outcome, Outcome Assessment (Health Care), Burns, Randomized Controlled Trials as Topic

Strengths and limitations of this study:

- This review is a comprehensive and systematic search for all clinical outcomes reported in randomised controlled trialss of burn care between and including 2012 and 2016.
- There is a detailed analysis of all reported outcomes and timing of outcome assessment.
- A multi-disciplinary team including a patient were involved in the study.
- Quality assessment of studies was not undertaken as the purpose of the review was to extract clinical outcomes alone and not to assess the effect of an intervention.

For peer review only

Introduction

Each year an estimated 2-300,000 people die from burn injuries globally(1). Millions more suffer from burn-related disabilities and disfigurements(2). These injuries have functional, psychological, social and economic effects on survivors and their families. There are multiple strategies for managing burn wounds and associated impact on patient physiology, with new care pathways and technology being introduced on a regular basis(3-5). The choice of treatment should be made using up-to-date, high quality scientific evidence (6, 7). Systematic reviews of randomised controlled trials (RCT) are regarded as the highest quality evidence (8-10). Despite increasing numbers of published RCTs in burn care, systematic reviews have not provided evidence to support many commonly used interventions or management strategies (11-13).

A well-designed RCT requires that outcomes are pre-specified. Evidence synthesis requires that these outcomes are consistent across RCTs in the same healthcare area (14). In the context of clinical trials, Williamson et al in the Core Outcome Measures in Effectiveness Trials (COMET) handbook, define an outcome as "a measurement or observation used to capture and assess the effect of treatment such as assessment of side effects (risk) or effectiveness (benefits)" (15). Chan adds a temporal element: "a variable measured at a specific time point to assess the efficacy or harm of an intervention"(16). If RCTs report outcomes that cannot be collated due to differences in choice, definition or timepoint of assessment, evidence synthesis will not be effective or efficient. There is no agreed minimum set of outcomes important to patients and professionals for reporting in burn care trials and problems with outcome reporting in burn care research have previously been suggested(17-19).

Pre-specifying outcomes requires research to determine and agree the most important outcomes for a clinical condition. If this is not undertaken, the outcomes reported may not reflect patients' or other stakeholders' needs, outcomes will vary between studies (outcome reporting heterogeneity) and it will be difficult to determine if authors have reported all the outcomes they measured (outcome reporting bias) (20, 21). Choosing the most important outcomes to measure in burn care is complex, as patients are a heterogeneous population, with variations in age, mechanism of injury, depth, site and size of burn (22, 23). The time frame at which outcomes are measured may also determine the types of outcomes assessed. Outcomes reported in clinical trials during the acute treatment phase include healing time, skin-graft loss, infection rates and NHS costs (24-27). Longer-term reported outcomes relate to functional, cosmetic and psychological issues (28).

For peer review only - http://bmjopen.bmj.com/site/about/guidelines.xhtml

To date, there has been no formal investigation into outcome reporting in trials of burn care. The purpose of this study is to examine clinical outcome reporting in burn care research, to consider the types, definitions and timing of outcomes measured and to consider the need for a Core Outcome Set in this field.

Methods

This review is focused on clinical observer-reported outcomes in RCTs assessing the impact of interventions in burn care. It adhered to a pre-specified protocol and the Preferred Reporting Items for Systematic Reviews and Meta-Analyses (PRISMA) statement(29). It was registered with the PROSPERO international prospective register of systematic reviews (ID CRD42017060908).

Study Eligibility

Studies were included if they met the following:

Types of studies: We included full text RCTs along with RCT protocols and pilot studies. The study design was limited to RCTs, as any final core outcome set will be used for RCT reporting(29). We excluded protocols and pilot studies if the full RCT had been published within the selected time period. We also excluded conference proceedings and abstracts, non-English language publications and studies not involving human subjects.

Types of participants: We included studies recording outcomes from patients of any age with a cutaneous burn of any type or size, determined by either clinician evaluation or objective assessment, or both, which required treatment in any health care facility. Studies where the population consisted of patients with combined thermal and mechanical injuries were only included if it was possible to separate out the burn care outcomes. Trials studying patients with pure carbon monoxide poisoning, chemical ocular or caustic oesophageal burns were excluded, as the former does not involve a burn and the latter have different aetiology and management to cutaneous burns.

Type of interventions: Any surgical or non-surgical burn care intervention with any appropriate *comparator.*

Types of outcomes: defined as the exact terms used in a published trial Abstract, Methods or Results including tables and figures for any observer-reported clinical endpoint. These included physiological, metabolic or adverse or mortality events measured by researchers and relevant to patients' recovery and long-term well-being after burn care (30). Trials assessing quality of life were only included if the data were observer-reported.

Identification of studies

For peer review only

Electronic searches of Ovid MEDLINE, Ovid EMBASE, Web of Science and The Cochrane Library were searched from 1st January 2012 to December 31st 2016 for RCTs related to burn care using medical subject heading and free text terms including 'burn', 'scald' 'thermal injury' and 'RCT'. This period was chosen so that the outcomes extracted, reflected use in trials relating to modern burn care. Limiting the review to five years allowed us to balance workload against the likelihood of selecting enough trials fulfilling inclusion criteria to demonstrate whether heterogeneity of outcome reporting was present in burn care research. The thesaurus vocabulary of each database was used to adapt the search terms. The search strategy for Ovid MEDLINE is included in a previous publication and in Appendix A(29).

Study selection process

Prior to both abstract and full-text screening, all review authors underwent training to ensure a comparable understanding of the purpose of the review and the eligibility criteria. The reference management software EndNote (Endnote 6, Clarivate Analytics, Boston; available at http://endnote.com/) was used to compile all titles derived from the initial searches, with duplicates removed for the review authors to screen titles and abstracts against the eligibility criteria. Screening of titles and abstracts was completed independently, then in duplicate by two authors (AY, AD) with experience in systematic review methodology. All screening disagreements were discussed, with any outstanding disagreements resolved by an independent reviewer (JB). Any studies appearing to meet the inclusion criteria based on the abstract were retrieved as full-text articles. Two reviewers then read the full-text articles in their entirety to assess for eligibility, with decisions on inclusion and exclusion recorded (Figure 1 for flow diagram). Reasons for exclusion were ordered hierarchically (Table 1) and applied to each full text. The highest reason for exclusion met by a paper was recorded as its reason for exclusion. Any disagreements were discussed with another author (JB).

Quality Assessment.

The aim of this study was to comprehensively document any variation in clinical outcomes selected, defined, measured and reported in burn care RCTs and not to synthesise data about the effect of interventions. Inclusion of all trials was necessary to demonstrate if a variation in outcome reporting was present across trials, regardless of quality of methodology of the trial. We therefore decided not to undertake a quality assessment of studies because it was not relevant to the data being recorded in this review; simply the nature and description of the unique outcomes reported in each study.

Data Extraction

For peer review only - http://bmjopen.bmj.com/site/about/guidelines.xhtml

For peer review only

Data were extracted into a standardised data extraction sheet (Microsoft Excel). This included study author, country or countries recruiting (categorised into the United Nations six regions (31)), publication year, number of sites and number of participants recruited per trial, design (full RCT, pilot, protocol) and intervention tested. For protocols, the planned participant inclusion criteria and sample size were extracted.

No distinction was made between primary or secondary outcomes, although this was noted and is part of a separate project. All outcomes were extracted verbatim, with 20% of the extracted data verified by a second reviewer. True duplicates, spelled and worded the same, were deleted. As a second process, two reviewers (a clinician and researcher) discussed all verbatim outcomes to assess duplicates in meaning but spelled or worded in a slightly different manner; such as length of time in hospital and number of days in hospital, platelet level and levels, and serum IL-10 and IL-10 in blood. These were named as one outcome with wording chosen by the reviewers and the others deleted as duplicates. The remaining outcomes were therefore all different in meaning. Any discrepancies were discussed with a senior researcher (JB). The number of outcomes per trial and the variation of outcomes between trials was recorded.

The time after injury that outcomes were measured were noted separately in order to a) assess the heterogeneity in outcome measurement timing and b) to understand at what stage after injury the effects of the intervention were being assessed. If a single outcome was assessed at different timepoints, *all* assessment timings were recorded. Data extraction for the timing of outcome reporting from 10% of trials was undertaken independently by another researcher. Timings of outcome assessment were categorised into time periods; ≤ 1 month, > 1 month and ≤ 3 months, > 3 months and ≤ 6 months after injury, > 6 months and ≤ 1 year and > 1 year and ≤ 3 years, and > 3 years. We reported two other outcome time periods; those assessed during acute hospitalisation and during burn wound healing, as these were commonly reported in the literature with no proscribed timepoint. However, it was clear from the reported length of stay and healing data, that all these outcomes were assessed within six months of injury. The frequency of outcomes reported within each time period was recorded.

The data were tabulated so that each study was listed with study and population details along with outcomes measured. Outcomes were extracted from this spreadsheet into another, with duplicates removed as described above. Outcomes measuring the same healthcare issue but at different timepoints were noted as one outcome for the final set. These final unique outcomes were then grouped into domains.

Classification of outcomes into domains

Outcome domains are groups of similar outcomes. This organisation is necessary, as maintaining a large set of outcomes when a significant number are similar, would make any future classification of the outcomes in terms of importance, extremely challenging.

For peer review only - http://bmjopen.bmj.com/site/about/guidelines.xhtml

Outcomes were classified into domains in a three-stage iterative approach. In stage one, four researchers (a clinically trained burn care researcher, a burn research associate and two senior research nurses experienced in burn care) independently reviewed the list of outcomes and attributed a potential domain to each one using their own terms. In stage two, the researchers met to review the domains and agreed i) appropriate groupings of outcomes into domains and ii) an appropriate name for each domain. Rules for attribution of outcomes to domains were recorded in a coding log to ensure consistency. In stage three, a patient representative reviewed the outcomes and their attributed domains to check for clarity of domain name, and that the outcomes under each domain were appropriately attributed. A final meeting with an experienced outcome researcher was held to finalise outcomes and domains. The use of a published classification system was not undertaken as none appeared to allow the flexibility or fit to the types of outcomes reported in burn care trials(32, 33).

The results described below indicate the characteristics of the reported studies and provide detail on heterogeneity of outcome reporting between studies, outcome definitions, timepoints and outcome domains.

Patient Involvement: The need for a burn care Core Outcome Set project was conceived following discussions regarding clinical healthcare Key Performance Indicators with professionals and patients. The patients were vocal about outcomes important to them which they felt were overlooked by professionals, such as pain. The systematic review was discussed at regular project steering group meetings attended by three patients with burns and one parent of a child with burns. A patient with burns is a co-author and was involved with writing and editing of this article as well as with the naming of the outcome domains. Dissemination will be to the lay representatives of the steering group and will inform the Core Outcome Set study in which patients are actively involved.

Results

Included studies and study protocols:

The initial search strategy identified 3,110 studies. Following de-duplication, a total of 2,070 studies remained. Independent scrutiny of the titles and abstracts identified 306 potentially relevant articles for full text review. Of these, 158 studies did not meet our inclusion criteria and were excluded (PRISMA Flow Diagram Figure 1). Therefore, a total of 147 studies formed the basis of this study(24, 34-178).

Studies: Of the 147 studies (Table 2), 86.4% (127) were reports of full RCTs, 8.8% (13) were pilot studies and 4.8% (7) were study protocols. The number of studies published increased between 2012 and 2016, with 26 RCTs published in 2012 and 40 in 2016 (Table 3).

For peer review only - http://bmjopen.bmj.com/site/about/guidelines.xhtml

For peer review only

9,022 participants were recruited across the 140 studies (study protocols not included n=7). The number of patients recruited per trial ranged from 3 to 612 (median 50; IQR 30-88) for full RCTs and from 10 to 52 (median 21; IQR 16-28) for pilot studies. 50.4% of full RCTs recruited fewer than 50 participants (Table 3). The majority (89.7%) of studies recruited (or planned to recruit) participants on one site alone. Of the 10.2% (15) of studies that were multi-centre, nine (60%) undertook research at only two or three sites. Thirty-two countries from the six global regions recruited patients into the 147 RCTs (Table 3). The country that undertook the most studies was the USA with 22.4% (33), followed by Iran with 12.9% (19) and China with 10.9% (16) of published studies. Of the 32 countries, 59.3% (19) published only one trial in this time period. The most common trial interventions related to dressings and wound care 29.2% (43), followed by surgical technique 11.6% (17) and management of pain and itch 10.9% (Table 3).

Outcomes:

1,494 clinical outcomes were reported of which, after de-duplication, 955 different, unique outcomes remained. Of the 1,494 outcomes reported, 27.7% (421) were common across two studies or more. Of *these* outcomes, 50.3% (78) appear in only two trials and 84.5% appear in five trials or fewer. The number of outcomes reported per trial varied from one to 37 (median 9; IQR 8) (Table 4). No single outcome was reported across all 147 studies.

Outcome definition variation: Outcomes assessing the same healthcare issue were commonly defined differently. An example is burn wound healing which was defined in 166 different ways. Examples include: healing percentage at specified timepoints, incidence of complete wound healing, incidence of 30% wound healing and length of time until 50% epithelialisation of burn wound. Similar variation in definition of burn wound infection existed with 79 unique outcome definitions including: bacterial colonisation of burn wound, days of antibiotics, incidence of local infection, incidence of positive wound cultures, peri-wound redness, rate of bacterial clearance from wound and number of inflammatory cells in the wound.

Outcome timing variation: There were 2,743 outcomes measured if the same outcome measured at different timepoints across all the 147 RCTs are included; e.g. size of burn wound measured at one week and again at two weeks, were recorded as different outcomes for this exercise. Of these, 76.9% (2,109) were assessed at less than six months after injury, 16.6% (456) were measured after six months and before three years after injury, and only 5.1% (140) were measured at more than three years after injury (Figure 2). The timing of outcome measurement was not reported for 38 outcomes.

Outcome domains: The 955 *different* clinical outcomes were organised into 54 domains (groups of similar outcomes). Table 5 categorises the domains into over-arching categories and gives examples and total numbers of outcomes within each domain.

For peer review only - http://bmjopen.bmj.com/site/about/guidelines.xhtml

For peer review only

Discussion

This systematic review aimed to examine outcome reporting in RCTs in burn care. Of the 147 included studies, 1,494 outcomes were identified, with overlap in terminology, inconsistent definitions and variation in time after injury at which the outcomes were measured. Only 30% of the outcomes reported were included in more than one study. There was no single outcome reported across all 147 trials. Commonly-reported outcomes were defined differently between trials, such as burn wound healing which was defined in 166 different ways. Such heterogeneity of outcome reporting across trials will limit evidence synthesis and result in research wastage.

The findings in this review have been seen elsewhere in the burns-specific and other clinical literature. A Cochrane review of 30 RCTs concluded that it was impossible to draw conclusions about burn dressing effectiveness, as the trials evaluated a variety of clinical outcomes(18, 179). Over the same period as this review, nine Cochrane reviews have had direct relevance to the management of patients with burns(18, 180-187). None could draw firm conclusions due to methodological issues including heterogeneity of outcome reporting. Heterogeneity is found in the reporting of outcomes relating to critical care, neurological illness, breast reconstruction surgery, prostate cancer, hip and knee replacement, oesophagectomy surgery, low back pain and in cardiac arrest trials among others (188-195). Variation in the definitions of outcomes has also been found within published studies of other healthcare areas. A systematic review of 90 papers reporting wound infection after general surgical procedures identified 41 definitions for wound infection itself, including three published by expert groups (196). Similarly, a total of 56 definitions were identified from 97 studies reporting anastomotic leak rates after gastrointestinal surgery despite publication of a standard definition two years before the beginning of the review (197).

In this review we identified and agreed the grouping of the 955 unique outcomes into 54 outcome domains. There is no agreement between COS reviewers about how best to classify outcomes into domains. Williamson published a taxonomy of categorising outcome domains (198). Other authors have suggested different ways of doing this, all addressing different needs(32, 33, 199)). In the Williamson taxonomy, the authors state that of 99 COS studies, 21 applied their own approach to outcome classification and only six used an existing system. As we had identified a large number of different clinical burn outcomes and as the outcomes we extracted did not clearly fall within the Williamson taxonomy, we decided to use our own approach to domain classification. We used five multi-disciplinary researchers and a patient working independently, and subsequently together, to bring different views and as little bias as possible to the process.

For peer review only - http://bmjopen.bmj.com/site/about/guidelines.xhtml

For peer review only

A solution to the above described variation in outcome reporting across trials, is the development of a Core Outcome Set (COS)(21, 200, 201). A COS is a minimum set of the most important outcomes, agreed and recommended for measurement in all trials for a particular condition (31, 32). While not limiting choice, a COS will pre-specify a set of outcomes to ensure consistency of reporting and the ability to collate evidence into systematic reviews by allowing researchers to compare ‘like with like’ (33). Trials can still select additional outcomes and primary outcomes in addition to the minimum core set. This approach has been shown to improve the consistency of outcome reporting (202, 203). Although there is no COS for burn care, work was undertaken in 2008 to agree a set of burn outcome domains(198). However, the work was undertaken by a small group of clinicians, lacked patient involvement and reported little methodological detail (204). Considerable work to develop COS methodology has also been undertaken since this publication (205, 206). The COMET (Core Outcome Measures in Effectiveness Trials) Initiative disseminates resources for COS development and supports methodological developments in this area(207, 208). COMET recommends a four-step process to develop a COS: a) agreement of the scope, b) assessment of the need, c) development of a protocol and finally d) agreement of the Core Outcome Set(15). This systematic review has satisfied the first two phases for the development of a burn care COS. The final phase encompasses organising a comprehensive long-list of all potential outcomes into domains (of which the clinical domains for burn care are listed in Table 5) and prioritising these domains using a consensus process (209-211).

The strengths of this review are that the protocol and data extraction proforma were pre-specified and the literature search was systematic and comprehensive, including four major healthcare trial databases. To account for multi-disciplinary perspectives, two researchers, two clinicians and a patient were involved in the domain process. It is also novel because it is the first to demonstrate, in detail and using systematic methodology, the scale of the heterogeneity of outcome reporting in burn care research. Limitations include the exclusion of publications in languages other than English. However, international publications were included to reduce the risk of selection bias. The search was also time-limited which may have excluded outcomes from older studies. The reason for the time limitation was to identify research relevant to modern burn care. The search was also limited to trials reporting clinical outcomes. Other work is in progress to assess patient-reported outcomes in burn care research. This was a review undertaken systematically to a pre-specified protocol. However, a formal quality assessment of studies was not undertaken, as we were researching the reporting of outcomes and not attempting to analyse the effects of interventions. A COS for burn care research would address the issue of heterogeneity of outcome reporting between trials, lead to research that is more likely to measure relevant outcomes, enhance the value of burn care systematic reviews and reduce research waste.

Conclusion

We have shown that multiple different unique outcomes are reported in trials of burn care interventions. Different definitions are used to assess the same outcome issue and outcomes are measured at different time

For peer review only - http://bmjopen.bmj.com/site/about/guidelines.xhtml

points after injury. This heterogeneity and inconsistency in outcome reporting prevent effective evidence synthesis and limits the quality of evidence available for clinical decision-making. Our review demonstrates that until greater consistency is achieved in outcome reporting in trials, it is unlikely that clinicians will be able to synthesise evidence across studies to understand the effects of surgical and non-surgical treatments following burn injury. It is recommended that a burn care Core Outcome Set is developed to support the effective synthesis of trial data and allow more informed clinical decision-making for the benefit of patients.

Contributors: Dr Amber Young wrote the paper and conceived the project with the support of Professor Jane Blazeby.

Professor Blazeby and Dr Brookes edited and critically revised the article. Dr Anna Davies contributed to data extraction and edited the paper. Ms Sophie Bland assisted in the domain name choice and article editing and readability.

All authors have read and approved the manuscript.

Acknowledgements: We would like to thank Dr Jason Wasiak, Senior Research Fellow, University of Melbourne, Monash University, Medical School, for his invaluable help in critically editing the paper. We would like to also thank Ms Joanna Hooper, senior outreach librarian and the University Hospitals Bristol NHS Foundation Trust medical library for their help in accessing articles.

Funding: This article/paper/report presents independent research funded by the National Institute for Health Research (NIHR) Doctoral Research Fellowship DRF-2016-09-031. The views expressed are those of the author(s) and not necessarily those of the NHS, the NIHR or the Department of Health. JB is part-funded by the Medical Research Council ConDuCT-II Hub (Collaboration and innovation for Difficult and Complex randomised controlled Trials In Invasive procedures—MR/K025643/1). The study was also supported by the NIHR Biomedical Research Centre at the University Hospitals Bristol NHS Foundation Trust and the University of Bristol.

PROSPERO number: ID CRD42017060908

Competing interests: JB is an NIHR Senior Investigator. All other authors declare no competing interests.

Ethics approval: South West Frenchay Research Ethics Committee (ref: 17/SW/0025
IRAS 221625).

Data Sharing: No further data are available.

For peer review only

Legends for Figures:

Figure 1: PRISMA Flow Diagram

Figure 2: Reported timepoint at which outcome was assessed

Table 1. Hierarchy of exclusion

1. Duplicate
2. Not published between Jan 1st, 2012 until Dec 31st, 2016.
3. Not written in English
4. Not relating to burns or burn care alone.
5. Population is non-human subjects (e.g. animal studies, modelling)
6. Abstract with no full text given
7. Study is not an RCT testing an intervention on burn care
8. Laboratory based, not carried out in clinical setting e.g. exudate or blood samples from humans tested within laboratory.
9. Systematic review paper, commentary paper.
10. Reports only patient-reported outcomes
11. Volunteer study
12. Oesophageal burns
13. Ocular burns
14. Anaesthetic / sedation technique
15. Diagnostic test trial
16. Unavailable anywhere
17. Follow-up studies

For peer review only

Table 2:

Trial title	First Author	Year of publication
1. Comparison of silver nylon wound dressing and silver sulfadiazine in partial burn wound therapy. (34)	Abedini F	2012
2. Healing of burn wounds by topical treatment: A randomized controlled comparison between silver sulfadiazine and nano-crystalline silver. (35)	Adhya A	2015
3. An analysis of deep vein thrombosis in burn patients (Part 1): Comparison of D-dimer and Doppler ultrasound as screening tools. (36)	Ahuja R	2016
4. An analysis of deep vein thrombosis in burn patients (part II): A randomized and controlled study of thrombo-prophylaxis with low molecular weight heparin. (37)	Ahuja R	2016
5. A four arm, double blind, randomized and placebo-controlled study of pregabalin in the management of post-burn pruritus. (38)	Ahuja R	2012
6. Propranolol attenuates haemorrhage and accelerates wound healing in severely burned adults. (39)	Ali A	2015
7. Aerobic exercise training in modulation of aerobic physical fitness and balance of burned patients. (40)	Ali Z	2015
8. Silk sericin ameliorates wound healing and its clinical efficacy in burn wounds. (41)	Aramwit P	2013
9. A Randomized Controlled Trial Comparing Endoscopic-Assisted Versus Open Neck Tissue Expander Placement in Reconstruction of Post-Burn Facial Scar Deformities. (42)	As'adi K	2016

For peer review only - http://bmjopen.bmj.com/site/about/guidelines.xhtml

10.	A prospective, randomised study of a novel transforming methacrylate dressing compared with a silver-containing sodium carboxymethylcellulose dressing on partial-thickness skin graft donor sites in burn patients. (43)	Assadian O	2013
11.	Multimodal quantitative analysis of early pulsed-dye laser treatment of scars at a pediatric burn hospital. (44)	Bailey J	2012
12.	Early fluid resuscitation with hydroxyethyl starch 130/0.4 (6%) in severe burn injury: a randomized, controlled, double-blind clinical trial. (45)	Bechir M	2013
13.	A prospective randomized trial comparing silver sulfadiazine cream with a water-soluble poly-antimicrobial gel in partial-thickness burn wounds. (46)	Black J	2015
14.	Clinical effectiveness of dermal substitution in burns by topical negative pressure: a multi-center randomized controlled trial. (47)	Bloeman M	2012
15.	Effect of subcutaneous epinephrine/saline/local anesthetic versus saline-only injection on split-thickness skin graft donor site perfusion, healing, and pain. (48)	Blome Eberwein S	2013
16.	A randomized controlled study of silver-based burns dressing in a pediatric emergency department. (49)	Brown M	2016
17.	Cost-Effectiveness of a Nonpharmacological Intervention in Pediatric Burn Care. (50)	Brown N	2015
18.	Play and heal: randomized controlled trial of DittoTM intervention efficacy on improving re-epithelialization in pediatric burns. (51)	Brown N	2013
19.	The implementation and evaluation of therapeutic touch in burn patients: an instructive experience of conducting a scientific study within a non-academic nursing setting.	Busch M	2012
20.	Prophylactic sequential bronchoscopy after inhalation injury: results from a three-year prospective randomized trial. (52)	Carr J	2013
21.	Burns injury in children: is antibiotic prophylaxis recommended? (53)	Chahed J	2014
22.	A randomized controlled trial to compare the effects of liquid versus powdered recombinant human growth hormone in treating patients with severe burns. (54)	Chen G	2016
23.	The Effect of Continuous Sedation Therapy on Immunomodulation, Plasma Levels of Antioxidants, and Indicators of Tissue Repair in Post-Burn Sepsis Patients. (55)	Chen Li	2015
24.	Application of acellular dermal xenografts in full-thickness skin burns. (56)	Chen X	2013
25.	Effectiveness of medical hypnosis for pain reduction and faster wound healing in pediatric acute burn injury: study protocol for a randomized controlled trial. (57)	Chester S	2016
26.	Safety of recombinant human granulocyte-macrophage colony-stimulating factor in healing pediatric severe burns. (58)	Chi Y	2015
27.	Comparison of three cooling methods for burn patients: A randomized clinical trial. (59)	Cho Y	2016
28.	The effect of burn rehabilitation massage therapy on hypertrophic scar after burn: a randomized controlled trial. (60)	Cho YS	2014
29.	Effect of extracorporeal shock wave therapy on scar pain in burn patients: A prospective, randomized, single-blind, placebo-controlled study. (61)	Cho YS	2016
30.	Characterization of early thermal burns and the effects of hyperbaric oxygen treatment: a pilot study. (62)	Chong SJ	2013
31.	Effects of different duration exercise programs in children with severe burns. (63)	Clayton RP	2016
32.	The effect of healing touch on sleep patterns of pediatric burn patients. (64)	Cone L	2014
33.	Effect of N-acetylcysteine treatment on oxidative stress and inflammation after severe burn. (65)	Csontos C	2012
34.	The effects of intravenous glutamine supplementation in severely burned, multiple traumatized patients. (66)	Cucerean-Badica	2013
35.	A comparison between occlusive and exposure dressing in the management of burn wound	Dallal MMS	2016
36.	Evaluation of the "Early" Use of Albumin in Children with Extensive Burns: A Randomized Controlled Trial	Dittrich MH	2016
37.	Interim pressure garment therapy (4-6 mmHg) and its effect on donor site healing in burn patients: study protocol for a randomised controlled trial. (67)	Donovan M	2016
38.	Effect of whole body vibration on leg muscle strength after healed burns: a randomized controlled trial. (68)	Ebid AA	2012
39.	Effect of isokinetic training on muscle strength, size and gait after healed pediatric burn: a randomized controlled study. (69)	Ebid AA	2014
40.	Effect of 12-week isokinetic training on muscle strength in adult with healed thermal burn. (70)	Ebid AA	2012
41.	Effects of whole-body vibration exercise on bone mineral content and density in thermally injured children. (71)	Edionwe J	2016
42.	Efficacy of platelet rich plasma application in comparison to conventional dressing therapy in partial thickness burn wound. (72)	Ehmer al Ibran	2014
43.	Effect of probiotic administration in the therapy of pediatric thermal burn. (73)	El-ghazely MH	2016
44.	Heparin/N-acetylcysteine: an adjuvant in the management of burn inhalation injury: a study of different doses. (74)	Elsharnouby NM	2014
45.	The effect of levamisole on mortality rate among patients with severe burn injuries. (75)	Fatemi MJ	2013
46.	Impact of stress-induced diabetes on outcomes in severely burned children. (76)	Finnerty CC	2014
47.	Outcome of Burns Treated With Autologous Cultured Proliferating Epidermal Cells: A Prospective Randomized Multi-center Intra-patient Comparative Trial. (77)	Gardien KL	2014
48.	Randomized controlled trial of three burns dressings for partial thickness burns in children. (78)	Gee Kee EL	2015
49.	Topical petrolatum gel alone versus topical silver sulfadiazine with standard gauze dressings for the treatment of superficial partial thickness burns in adults: a randomized controlled trial. (79)	Genuino GAS	2014
50.	HEPBURN - investigating the efficacy and safety of nebulized heparin versus placebo in burn patients with inhalation trauma: study protocol for a multi-center randomized controlled trial. (80)	Glas GJ	2014
51.	A multi-center study on the regenerative effects of erythropoietin in burn and scalding injuries: study protocol for a randomized controlled trial. (82)	Gunter CI	2013
52.	Early rehabilitative exercise training in the recovery from pediatric burn. (83)	Hardee JP	2014
53.	Quality of pediatric second-degree burn wound scars following the application of basic fibroblast growth factor: results of a randomized, controlled pilot study. (84)	Hayashida K	2012
54.	Long-term propranolol use in severely burned pediatric patients: a randomized controlled study. (85)	Herndon DN	2012
55.	Reversal of growth arrest with the combined administration of oxandrolone and propranolol in severely burned children. (86)	Herndon DN	2016

For peer review only - http://bmjopen.bmj.com/site/about/guidelines.xhtml

56.	Cost-Effectiveness of Laser Doppler Imaging in Burn Care in The Netherlands: A Randomized Controlled Trial. (87)	Hop MJ	2016
57.	Effect of music intervention on burn patients' pain and anxiety during dressing changes. (88)	Hsu K	2016
58.	Low dose of glucocorticoid decreases the incidence of complications in severely burned patients by attenuating systemic inflammation. (89)	Huang G	2015
59.	An assessment of early Child Life Therapy pain and anxiety management: A prospective randomised controlled trial. (90)	Hyland E	2015
60.	Prospective, randomised controlled trial comparing Versajet™ hydrosurgery and conventional debridement of partial thickness paediatric burns. (91)	Hyland EJ	2015
61.	Construction of skin graft seams in burn patients: A prospective randomized double-blinded study. (92)	Isaac K	2016
62.	Multi-axis shoulder abduction splint in acute burn rehabilitation: a randomized controlled pilot trial. (93)	Jang KU	2015
63.	Glucose control in severely burned patients using metformin: An interim safety and efficacy analysis of a phase II randomized controlled trial. (94)	Jeschke MG	2016
64.	The effect of ketoconazole on post-burn inflammation, hypermetabolism and clinical outcomes. (95)	Jeschke MG	2012
65.	The Effect of Distraction Technique on the Pain of Dressing Change among 3-6 Year-old Children. (96)	Kaheni S	2016
66.	Prospective randomize-controlled comparison between silicone plus herbal extract gel versus Aloe Vera gel for burn scar prophylaxis. (97)	Keorochana K	2015
67.	Effects of Enteral Glutamine Supplementation on Reduction of Infection in Adult Patients with Severe Burns. (98)	Kibor DK	2014
68.	Effects of sustained release growth hormone treatment during the rehabilitation of adult severe burn survivors. (99)	Kim J	2016
69.	Virtual reality for acute pain reduction in adolescents undergoing burn wound care: a prospective randomized controlled trial. (100)	Kipping B	2012
70.	The effects of splinting on shoulder function in adult burns. (101)	Kolmus AM	2012
71.	Prospective study on burns treated with Integra, a cellulose sponge and split thickness skin graft: comparative clinical and histological study--randomized controlled trial. (102)	Lagus H	2013
72.	Evaluation of an oxygen-diffusion dressing for accelerated healing of donor-site wounds. (103)	Lairet KF	2014
73.	Anti-inflammatory effect of taurine in burned patients. (104)	Lak S	2015
74.	A randomized controlled pilot study comparing aqueous cream with a beeswax and herbal oil cream in the provision of relief from postburn pruritis. (105)	Lewis PA	2012
75.	Human acellular dermal matrix allograft: A randomized, controlled human trial for the long-term evaluation of patients with extensive burns. (106)	Li X	2015
76.	Selective digestive decontamination attenuates organ dysfunction in critically ill burn patients. (107)	Lopez-Rodriguez L	2015
77.	Results of a prospective randomized controlled trial of early ambulation for patients with lower extremity autografts. (108)	Lorello DJ	2014
78.	Moist occlusive dressing (Aquacel(Â®) Ag) versus moist open dressing (MEBO(Â®)) in the management of partial-thickness facial burns: a comparative study in Ain Shams University. (109)	Mabrouk A	2012
79.	Enhancement of burn wounds healing by platelet dressing. (110)	Maghsoudi H	2013
80.	Effect of immune-enhancing diets on the outcomes of patients after major burns. (111)	Mahmoud WH	2014
81.	Silver-coated nylon dressing plus active DC microcurrent for healing of autogenous skin donor sites. (112)	Malin EW	2013
82.	The application of platelet-rich plasma in the treatment of deep dermal burns: A randomized, double-blind, intra-patient-controlled study. (113)	Marck RE	2016
83.	Clinical safety and efficacy of probiotic administration following burn injury. (114)	Mayes T	2015
84.	Three donor site dressings in pediatric split-thickness skin grafts: study protocol for a randomised controlled trial. (115)	McBride CA	2015
85.	Evaluation of who oral rehydration solution (ORS) and salt tablets in resuscitating adult patients with burns covering more than 15% of total body surface area (TBSA). (116)	Moghazy AM	2016
86.	Efficacy and adverse events of early high-frequency oscillatory ventilation in adult burn patients with acute respiratory distress syndrome. (117)	Mohamed SA	2016
87.	Effect of amniotic membrane on graft take in extremity burns. (118)	Mohammadi AA	2013
88.	Comparison of the application of allogeneic fibroblast and autologous mesh grafting with conventional method in the treatment of third-degree burns. (119)	Moravvej H	2016
89.	Effect of low-intensity laser on the neuropathic common peroneal nerve post burn. (120)	Mowafy ZM	2016
90.	Clinical Efficacy Test of Polyester Containing Herbal Extract Dressings in Burn Wound Healing. (121)	Muangman P	2016
91.	Effect of oral olive oil on healing of 10-20% total body surface area burn wounds in hospitalized patients. (123)	Najmi M	2015
92.	Double-blind, randomized, pilot study assessing the resolution of postburn pruritus. (124)	Nedelec B	2012
93.	Comparing outcomes of sheet grafting with 1:1 mesh grafting in patients with thermal burns: a randomized trial. (125)	Nikkah D	2014
94.	Comparison of hydrogel produced by radiation as applied at the research center (Yazd branch) with maxgel and routine dressing for second-degree burn repair in Yazd burn hospital. (126)	Noorbala MT	2016
95.	Effectiveness of cerium nitrate-silver sulfadiazine in the treatment of facial burns: a multi-center, randomized, controlled trial. (127)	Oen IMMH	2012
96.	Influences of purposeful activity versus rote exercise on improving pain and hand function in pediatric burn. (128)	Omar MTA	2012
97.	Botulinum toxin and burn induces contracture. (130)	Omranifard M	2016
98.	Results of a pilot multi-center genotype-based randomized placebo-controlled trial of propranolol to reduce pain after major thermal burn injury. (129)	Orrey DC	2015
99.	A proper enteral nutrition support improves sequential organ failure score and decreases length of stay in hospital in burned patients. (131)	Ostradrahimi A	2016
100.	Topical silver sulfadiazine vs collagenase ointment for the treatment of partial thickness burns in children: a prospective randomized trial. (132)	Ostlie DJ	2012

For peer review only

For peer review only - http://bmjopen.bmj.com/site/about/guidelines.xhtml

101. Prospective randomized phase II Trial of accelerated re-epithelialization of superficial second-degree burn wounds using extracorporeal shock wave therapy. (133)	Ottomann C	2012
102. A randomized and controlled multi-center prospective study of the Chinese medicinal compound Fufang Xuelian Burn Ointment for the treatment of superficial and deep second-degree burn wounds. (134)	Ouyang J	2014
103. Prospective comparison of packed red blood cell-to-fresh frozen plasma transfusion ratio of 4: 1 versus 1: 1 during acute massive burn excision. (135)	Palmieri T	2012
104. A herbal cream consisting of Aloe Vera, Lavandulastoechas, and Pelargonium roseum as an alternative for silver sulfadiazine in burn management. (136)	Panahi Y	2012
105. Interactive gaming consoles reduced pain during acute minor burn rehabilitation: A randomized, pilot trial. (137)	Parker M	2016
106. A Pilot Prospective Randomized Control Trial Comparing Exercises Using Videogame Therapy to Standard Physical Therapy: 6 Months Follow-Up. (138)	Parry I	2015
107. An open, prospective, randomized pilot investigation evaluating pain with the use of a soft silicone wound contact layer vs bridal veil and staples on split thickness skin grafts as a primary dressing. (139)	Patton ML	2013
108. Effects of community-based exercise in children with severe burns: A randomized trial. (140)	Pena R	2015
109. Effects of propranolol and exercise training in children with severe burns. (141)	Porro LJ	2013
110. Five-year outcomes after oxandrolone administration in severely burned children: a randomized clinical trial of safety and efficacy. (142)	Porro LJ	2012
111. Clinical effectiveness, quality of life and cost-effectiveness of Flaminal versus Flamazine in the treatment of partial thickness burns: study protocol for a randomized controlled trial. (144)	Rashaan ZM	2016
112. Five-Year Outcomes after Long-Term Oxandrolone Administration in Severely Burned Children: A Randomized Clinical Trial. (145)	Reeves PT	2016
113. A novel rapid and selective enzymatic debridement agent for burn wound management: a multi-center RCT. (146)	Rosenburg L	2013
114. Effects of cholecalciferol supplementation and optimized calcium intakes on vitamin D status, muscle strength and bone health: a one-year pilot randomized controlled trial in adults with severe burns. (147)	Rousseau AF	2014
115. Evaluation of Amniotic Membrane Effectiveness in Skin Graft Donor Site Dressing in Burn Patients. (148)	Salehi SH	2015
116. A feasibility study assessing cortical plasticity in chronic neuropathic pain following burn injury. (143)	Santos Portilla A	2013
117. Perioperative treatment algorithm for bleeding burn patients reduces allogeneic blood product requirements. (149)	Schaden E	2012
118. A prospective clinical trial comparing Biobrane, Dressilk, and PolyMem dressings on partial-thickness skin graft donor sites. (150)	Schulz A	2016
119. Effectiveness of Aloe Vera gel compared with 1% silver sulphadiazine cream as burn wound dressing in second degree burns. (151)	Shahzad M	2013
120. The comparison between modified kligman formulation versus kligman formulation and intense pulsed light in the treatment of the post-burn hyperpigmentation. (152)	Siadat A	2016
121. A comparative study of spray keratinocytes and autologous meshed split-thickness skin graft in the treatment of acute burn injuries. (154)	Sood R	2015
122. Long-Term Administration of Oxandrolone Improves Lung Function in Pediatric Burned Patients. (155)	Sousse LE	2016
123. An open, parallel, randomized, comparative, multicenter investigation evaluating the efficacy and tolerability of Mepilex Ag versus silver sulfadiazine in the treatment of deep partial-thickness burn injuries. (156)	Tang H	2015
124. Non-ablative fractional laser provides long-term improvement of mature burn scars - A randomized controlled trial with histological assessment. (157)	Taudorf EH	2015
125. Fluid therapy lidco controlled trial - Optimization of volume resuscitation of extensively burned patients through noninvasive continuous real-time hemodynamic monitoring LiDCO. (158)	Tokarik M	2013
126. Burn donor site dressing using melolin and flexigrid versus conventional dressing. (159)	Vejdan SA	2015
127. Laser Doppler imaging as a tool in the burn wound treatment protocol. (160)	Venclauskiene A	2014
128. Low-dose hydrocortisone reduces norepinephrine duration in severe burn patients: a randomized clinical trial. (161)	Venet F	2015
129. A Comparative Study of Paediatric Thermal Burns Treated with Topical Heparin and Without Heparin. (162)	Venkatachalapathy TS	2014
130. Aquacel() Ag dressing versus ActicoatTM dressing in partial thickness burns: a prospective, randomized, controlled study in 100 patients. Part 1: burn wound healing. (24)	Verbelen J	2013
131. Skin stretching for primary closure of acute burn wounds. (163)	Verhaegen PDHM	2014
132. Xbox KinectTM based rehabilitation as a feasible adjunct for minor upper limb burns rehabilitation: A pilot RCT. (164)	Voon K	2016
133. Local application of low-dose insulin in improving wound healing after deep burn surgery. (165)	Wang C	2016
134. Gabapentin is ineffective as an analgesic adjunct in the immediate postburn period. (166)	Wibbenmeyer L	2014
135. A prospective randomised clinical pilot study to compare the effectiveness of Biobrane (R) synthetic wound dressing, with or without autologous cell suspension, to the local standard treatment regimen in paediatric scald injuries. (167)	Wood F	2012
136. Effective symptomatic treatment for severe and intractable pruritus associated with severe burn-induced hypertrophic scars: A prospective, multicenter, controlled trial. (168)	Wu J	2016
137. Propranolol reduces cardiac index but does not adversely affect peripheral perfusion in severely burned children. (169)	Wurzer P	2016
138. A new method of microskin autografting with a Vaseline-based moisture dressing on granulation tissue. (170)	Xiao H	2014
139. Recombinant human granulocyte-macrophage colony-stimulating factor hydrogel promotes healing of deep partial thickness burn wounds. (171)	Yan H	2012
140. A comparative study of the dressings silver sulfadiazine and Aquacel Ag in the management of superficial partial-thickness burns. (172)	Yarboro D	2013
141. A clinical trial designed to evaluate the safety and effectiveness of a thermosensitive hydrogel-type cultured epidermal allograft for deep second-degree burns. (173)	Yim H	2014

For peer review only

For peer review only - http://bmjopen.bmj.com/site/about/guidelines.xhtml

142. Study of the use of recombinant human granulocyte-macrophage colony-stimulating factor hydrogel externally to treat residual wounds of extensive deep partial-thickness burn. (174)	Yuan L	2015
143. Effect of Olea ointment and Acetate Mafenide on burn wounds - A randomized clinical trial. (175)	Zahmatkesh M	2015
144. Effects of puerarin on the inflammatory role of burn-related procedural pain mediated by P2X7 receptors. (176)	Zhang J	2013
145. Effects of early enteral nutrition on the gastrointestinal motility and intestinal mucosal barrier of patients with burn-induced invasive fungal infection. (81)	Zhang Y	2016
146. Maximizing the safety of glycerol preserved human amniotic membrane as a biological dressing. (177)	Zidan SM	2015
147. Therapeutic Value of Blood Purification and Prognostic Utilities of Early Serum Procalcitonin, C Reactive Protein, and Brain Natriuretic Peptide Levels in Severely Burned Patients with Sepsis. (178)	Zu H	2015

Table 3: RCT detail

	**Studies**
Number of RCTs	127/147 (86.4%)
Number of Pilot studies	13/147 (8.8%)
Number of RCT protocols	7/147 (4.8%)
**World region for recruitment:**	
- Asia	54/147 (36.7%)
- North America	36/147 (24.5%)
- Europe	26/147 (17.7%)
- Africa	15/147 (8.5%)
- Latin America	1/147 (0.7%)
- Australasia	15/147 (8.5%)
**Year published:**	
- 2012	26/147 (17.8%)
- 2013	24/147 (16.3%)
- 2014	24/147 (16.3%)
- 2015	33/147 (22.4%)
- 2016	40/147 (27.2%)
**Number of sites:**	
- 1	132/147 (89.8%)
- 2-3	9/147 (6.1%)
- 4-5	2/147 (1.4%)
- > 5	4/147 (2.7%)
**Number of participants in full RCTs:**	
- ≤ 10	4/127 (3.1%)
- 11-50	62/127 (48.8%)
- 51-100	39/127 (30.7%)
- 101-150	11/127 (8.7%)
- >150	11/127 (8.7%)
**Participants recruited:**	
- ≤ 18 years	24/147 (16.3%)
- > 18 years	59/147 (40.1%)
- Mixed age range	25/147 (17.0%)
- Not stated	34/147 (23.1%)
- N/A (protocol)[c]	5/147 (3.4%)
**Type of intervention:**	
- Dressings and wound care	38/147 (25.9%)
- Surgical technique	19/147 (12.9%)
- Treatment of pain or itch[a]	16/147 (10.9%)
- Impact of exercise and rehabilitation	13/147 (8.8%)
- Intensive care management	10/147 (6.8%)
- Treatment of hypermetabolism	8/147 (5.4%)
- Nutrition	8/147 (5.4%)
- Scar management	7/147 (4.8%)
- Treatment of inhalational injury	3/147 (2.0%)
- Use of topical rHGM	3/147 (2.0%)
- Use of rHGH	3/147 (2.0%)
- Sugar management	2/148 (2.0%)
- Treatment of infection	2/147 (1.4%)

For peer review only

- Treatment of DVT	2/147 (1.4%)
- Blood management	2/147 (1.4%)
- Extracorporeal shock wave therapy	2/147 (1.4%)
- Platelet rich plasma use	2/147 (1.4%)
- Others[b]	7/147 (5.4%)

[a]Inc. distraction for dressing changes
[b]Inc. levamisole, hyperbaric oxygen, fibroblast growth factor, oral calcium use, ketoconazole, low intensity laser

Table 4: Reported outcomes

**Number of outcomes per study:**	
- 1	4/147 (27.2%)
- 2-5	34/147 (23.1%)
- 6-10	53/147 (36.1%)
- 11-20	41/147 (27.9%)
- >20	15/147 (10.2%)

For peer review only

Table 5: Outcome category, domains and examples of outcomes

Outcome Category	Outcome Domain	Outcome Examples	Nos. of unique outcomes per domain
Patient-reported	Ability to carry out daily tasks	Functional level of independence	1
	Anxiety about medical procedures and appointments	Pain anxiety<br>Anxiety before dressing changes	4
	Generalised anxiety	General anxiety	1
	Appearance	Facial symmetry<br>Overall scar appearance	3
	Blister fluid	Amount of exudate	3
	Burden of care	Frequency of dressing changes<br>Time taken for daily wound cleaning	7
	Comfort of dressings	Dressing comfort	1
	Psychological wellbeing	Improvement in well-being	1
	Mental ability		2
	Quality and quantity of sleep	Quantity of sleep<br>Incidence of sleep disturbance	17
	Effect of scar on movement (contractures)	Cognitive performance	3
	Return to work/school or previous function	Return to work or previous function	1
	Burn wound pain	Wound pain intensity at baseline<br>Pain tolerance	29
	Donor site pain	Donor site pain at rest	6

For peer review only - http://bmjopen.bmj.com/site/about/guidelines.xhtml

		Donor site pain while walking	
	Pain during procedures	Wound pain at dressing changes<br>Pain during hydrotherapy	14
	Scar pain	Functional scar pain<br>Incidence of neuropathic pain	13
	Itch	Baseline pruritus<br>Itch severity reduction	24
Pathophysiological	Ability to fight infection	Change in IgA<br>IL-1beta in blood<br>Serum interferon gamma levels	36
	Body weight maintenance	Incidence of weight loss<br>Body weight decrease from baseline	26
	Bone strength	Bone mineral density<br>Incidence of osteoporosis	30
	Breathing and lungs	Forced expiratory volume in 1 second<br>Functional residual capacity	27
	Donor site problems after healing	Donor site pigmentation<br>Sensation of donor site	24
	Effect of burn on genes	Gene expression patterns	1
	Effect of burn on how the body uses energy	Change in percentage of predicted resting energy expenditure	2
	Effect on heart and blood circulation	Incidence of cardiomegaly<br>Number of patients requiring noradrenaline	28
	Fitness	Max. aerobic capacity<br>Exercise max. minute ventilation	12
	Growth in children	Duration of growth arrest<br>Percentage change in height	10
	How well muscles work	Facial mimic function<br>Change in muscle function	9
	Mobility	Stride length<br>Knee range of motion	22
	Kidney function	Incidence of acute kidney injury<br>Requirement for renal replacement therapy	17
	Liver function	Hepatic function<br>Ammonia levels	11
	Medical tests to indicate how unwell a patient is	Albumin level<br>Change in Ph	84
	More than one organ failing (multi-organ failure)	Incidence of multi organ failure<br>Percentage of patients with organ dysfunction	7
	Muscle strength	Knee extensor strength<br>Hamstring strength adjusted for body weight	30
	Stomach and bowel function	Days of diarrhoea<br>Incidence of abdominal distension	13
	Burn wound healing	Burn wound area at timepoints<br>Days until wound closure	166

For peer review only

	Donor site healing	Donor site healing to 90%<br>Time to donor site re-epithelialisation	9
Complications	Complications of drug treatment	Adverse drug reactions<br>Allergic dermatitis	52
	Blood product transfusion	Blood transfused per kg during hospitalisation<br>Total volume FFP transfused	11
	Burn wound infection	Wound bacterial colonisation<br>Wound contamination post-operatively	80
	Death from burn injury	Mortality related to burn size	1
	Death from any cause	Overall mortality<br>In-hospital mortality	14
	Effects of fluid from a drip	Incidence of fluid creep<br>Net fluid balance at specified times	17
	Infections other than burn wound infection	Incidence of central catheter related infections<br>Pulmonary infection	7
	Sepsis	Days of sepsis<br>Incidence of positive blood cultures	7
Scar-related	Scar colour	Erythema index<br>Scar melanin levels	25
	Scar texture	Scar height<br>Change in scar distensibility	47
	Scar size	Scar surface area	1
	Treatment for scars	Numbers of patients assessed for scar management<br>Numbers of patients needing scar management	2
Healthcare-related	Costs of treatment for NHS/Hospital	Costs of analgesics for dressing changes<br>Pressure garment costs	14
	Length of hospital stay	Length of stay adjusted for burn size<br>Days in hospital	7
	Length of stay in intensive care unit	Length of ICU stay	3
	Length of time on life support machine	Duration of mechanical ventilation	1
	Use of medicines to treat symptoms	Pain relief required during dressing changes<br>Opioid consumption	12

For peer review only

References:

1. Mock C, Peck M, Peden M, Krug E, Organization WH. A WHO plan for burn prevention and care. Geneva: World Health Organization. 2008:3.

2. Peck M, Molnar J, Swart D. A global plan for burn prevention and care. Bulletin of the World Health Organization. 2009;87(10):802-3.

3. Atiyeh BS, Hayek SN, Gunn SW. New technologies for burn wound closure and healing—review of the literature. Burns. 2005;31(8):944-56.

4. Jeschke MG, Shahrokhi S, Finnerty CC, Branski LK, Dibildox M, Organization A, et al. Wound coverage technologies in burn care: established techniques. Journal of Burn Care & Research. 2018;39(3):313-8.

5. Rowan MP, Cancio LC, Elster EA, Burmeister DM, Rose LF, Natesan S, et al. Burn wound healing and treatment: review and advancements. Critical care. 2015;19(1):243.

6. Sackett DL, Rosenberg WM, Gray JM, Haynes RB, Richardson WS. Evidence based medicine: what it is and what it isn't. British Medical Journal Publishing Group; 1996.

7. Dickinson HD. Evidence-based decision-making: an argumentative approach. International Journal of Medical Informatics. 1998;51(2-3):71-81.

8. Every-Palmer S, Howick J. How evidence-based medicine is failing due to biased trials and selective publication. Journal of evaluation in clinical practice. 2014;20(6):908-14.

9. Muir Gray J. Evidence-based healthcare: how to make health policy and management decisions. London: Churchill Livingstone. 1997;53.

10. Centre for evidence based medicine. CEBM Levels of evidence 2011 [Available from: https://www.cebm.net/wp-content/uploads/2014/06/CEBM-Levels-of-Evidence-2.1.pdf.

11. Flores O, Stockton K, Roberts JA, Muller MJ, Paratz JD. The efficacy and safety of adrenergic blockade after burn injury: A systematic review and meta-analysis. J Trauma Acute Care Surg. 2016;80(1):146-55.

12. Avni T, Levcovich A, Ad-El DD, Leibovici L, Paul M. Prophylactic antibiotics for burns patients: systematic review and meta-analysis. Bmj. 2010;340:c241.

13. Aziz Z, Abu S, Chong N. A systematic review of silver-containing dressings and topical silver agents (used with dressings) for burn wounds. Burns. 2012;38(3):307-18.

14. Liberati A, Altman DG, Tetzlaff J, Mulrow C, Gøtzsche PC, Ioannidis JP, et al. The PRISMA statement for reporting systematic reviews and meta-analyses of studies that evaluate health care interventions: explanation and elaboration. PLoS medicine. 2009;6(7):e1000100.

15. Williamson PR, Altman DG, Bagley H, Barnes KL, Blazeby JM, Brookes ST, et al. The COMET handbook: version 1.0. Trials. 2017;18(3):280.

16. Chan A-W, Krleža-Jerić K, Schmid I, Altman DG. Outcome reporting bias in randomized trials funded by the Canadian Institutes of Health Research. Canadian Medical Association Journal. 2004;171(7):735-40.

17. Van Baar M, Essink-Bot M-L, Oen I, Dokter J, Boxma H, van Beeck EF. Functional outcome after burns: a review. Burns. 2006;32(1):1-9.

For peer review only - http://bmjopen.bmj.com/site/about/guidelines.xhtml

18. Wasiak J, Cleland H, Campbell F, Spinks A. Dressings for superficial and partial thickness burns. The Cochrane Library. 2013.

19. Lawrence JW, Mason ST, Schomer K, Klein MB. Epidemiology and impact of scarring after burn injury: a systematic review of the literature. Journal of burn care & research. 2012;33(1):136-46.

20. Sinha IP, Smyth RL, Williamson PR. Using the Delphi technique to determine which outcomes to measure in clinical trials: recommendations for the future based on a systematic review of existing studies. PLoS medicine. 2011;8(1):e1000393.

21. Williamson PR, Altman DG, Blazeby JM, Clarke M, Devane D, Gargon E, et al. Developing core outcome sets for clinical trials: issues to consider. Trials. 2012;13(1):132.

22. Klein MB, Goverman J, Hayden DL, Fagan SP, McDonald-Smith GP, Alexander AK, et al. Benchmarking outcomes in the critically injured burn patient. Annals of surgery. 2014;259(5):833.

23. Pereira C, Murphy K, Herndon D. Outcome measures in burn care: is mortality dead? Burns. 2004;30(8):761-71.

24. Verbelen J, Hoeksema H, Heyneman A, Pirayesh A, Monstrey S. Aquacel® Ag dressing versus Acticoat™ dressing in partial thickness burns: a prospective, randomized, controlled study in 100 patients. Part 1: Burn wound healing. Burns. 2014;40(3):416-27.

25. Hop MJ, Polinder S, Vlies CH, Middelkoop E, Baar ME. Costs of burn care: a systematic review. Wound repair and regeneration. 2014;22(4):436-50.

26. Azzopardi EA, Azzopardi E, Camilleri L, Villapalos J, Boyce DE, Dziewulski P, et al. Gram negative wound infection in hospitalised adult burn patients-systematic review and metanalysis. PloS one. 2014;9(4):e95042.

27. Griffiths HR, Thornton K, Clements CM, Burge T, Kay A, Young A. The cost of a hot drink scald. Burns. 2006;32(3):372-4.

28. Holavanahalli RK, Helm PA, Kowalske KJ. Long-term outcomes in patients surviving large burns: the musculoskeletal system. Journal of Burn Care & Research. 2016;37(4):243-54.

29. Young A, Brookes S, Rumsey N, Blazeby J. Agreement on what to measure in randomised controlled trials in burn care: study protocol for the development of a core outcome set. BMJ open. 2017;7(6):e017267.

30. Hopkins J, Howes N, Chalmers K, Savovic J, Whale K, Coulman K, et al. Outcome reporting in bariatric surgery: an in-depth analysis to inform the development of a core outcome set, the BARIACT S tudy. obesity reviews. 2015;16(1):88-106.

31. United Nations. [Available from: www.un.org/esa/population/publications/worldageing19502050/pdf/96annexii.pdf.

32. Smith V, Clarke M, Williamson P, Gargon E. Survey of new 2007 and 2011 Cochrane reviews found 37% of prespecified outcomes not reported. Journal of clinical epidemiology. 2015;68(3):237-45.

33. Davey J, Turner RM, Clarke MJ, Higgins JP. Characteristics of meta-analyses and their component studies in the Cochrane Database of Systematic Reviews: a cross-sectional, descriptive analysis. BMC medical research methodology. 2011;11(1):160.

34. Abedini F, Ahmadi A, Yavari A, Hosseini V, Mousavi S. Comparison of silver nylon wound dressing and silver sulfadiazine in partial burn wound therapy. International wound journal. 2013;10(5):573-8.

For peer review only - http://bmjopen.bmj.com/site/about/guidelines.xhtml

35. Adhya A, Bain J, Ray O, Hazra A, Adhikari S, Dutta G, et al. Healing of burn wounds by topical treatment: A randomized controlled comparison between silver sulfadiazine and nano-crystalline silver. Journal of basic and clinical pharmacy. 2014;6(1):29.

36. Ahuja RB, Bansal P, Pradhan GS, Subberwal M. An analysis of deep vein thrombosis in burn patients (Part 1): Comparison of D-dimer and Doppler ultrasound as screening tools. Burns. 2016;42(8):1686-92.

37. Ahuja RB, Bansal P, Pradhan GS, Subberwal M. An analysis of deep vein thrombosis in burn patients (part II): A randomized and controlled study of thrombo-prophylaxis with low molecular weight heparin. Burns. 2016;42(8):1693-8.

38. Ahuja RB, Gupta GK. A four arm, double blind, randomized and placebo controlled study of pregabalin in the management of post-burn pruritus. Burns. 2013;39(1):24-9.

39. Ali A, Herndon DN, Mamachen A, Hasan S, Andersen CR, Grogans R-J, et al. Propranolol attenuates hemorrhage and accelerates wound healing in severely burned adults. Critical care. 2015;19(1):217.

40. Ali ZMI, El-Refay BH, Ali RR. Aerobic exercise training in modulation of aerobic physical fitness and balance of burned patients. Journal of physical therapy science. 2015;27(3):585-9.

41. Aramwit P, Palapinyo S, Srichana T, Chottanapund S, Muangman P. Silk sericin ameliorates wound healing and its clinical efficacy in burn wounds. Archives of dermatological research. 2013;305(7):585-94.

42. As'adi K, Emami SA, Salehi SH, Shoar S. A randomized controlled trial comparing endoscopic-assisted versus open neck tissue expander placement in reconstruction of post-burn facial scar deformities. Aesthetic plastic surgery. 2016;40(4):526-34.

43. Assadian O, Arnoldo B, Purdue G, Burris A, Skrinjar E, Duschek N, et al. A prospective, randomised study of a novel transforming methacrylate dressing compared with a silver-containing sodium carboxymethylcellulose dressing on partial-thickness skin graft donor sites in burn patients. International wound journal. 2015;12(3):351-6.

44. Bailey JK, Burkes SA, Visscher MO, Whitestone J, Kagan RJ, Yakuboff KP, et al. Multimodal Quantitative Analysis of Early Pulsed-Dye Laser Treatment of Scars at a Pediatric Burn Hospital. Dermatologic Surgery. 2012;38(9):1490-6.

45. Béchir M, Puhan MA, Fasshauer M, Schuepbach RA, Stocker R, Neff TA. Early fluid resuscitation with hydroxyethyl starch 130/0.4 (6%) in severe burn injury: a randomized, controlled, double-blind clinical trial. Critical Care. 2013;17(6):R299.

46. Black JS, Drake DB. A prospective randomized trial comparing silver sulfadiazine cream with a water-soluble polyantimicrobial gel in partial-thickness burn wounds. Plastic Surgical Nursing. 2015;35(1):46-9.

47. Bloemen MC, Wal M, Verhaegen PD, Nieuwenhuis MK, Baar ME, Zuijlen PP, et al. Clinical effectiveness of dermal substitution in burns by topical negative pressure: a multicenter randomized controlled trial. Wound Repair and Regeneration. 2012;20(6):797-805.

48. Blome-Eberwein S, Abboud M, Lozano DD, Sharma R, Eid S, Gogal C. Effect of subcutaneous epinephrine/saline/local anesthetic versus saline-only injection on split-thickness skin graft donor site perfusion, healing, and pain. Journal of Burn Care & Research. 2013;34(2):e80-e6.

49. Brown MR, Dalziel SR, Herd E, Johnson K, Wong She R, Shepherd M. A randomized controlled study of silver-based burns dressing in a pediatric emergency department. Journal of Burn Care & Research. 2016;37(4):e340-e7.

For peer review only - http://bmjopen.bmj.com/site/about/guidelines.xhtml

50. Brown NJ, David M, Cuttle L, Kimble RM, Rodger S, Higashi H. Cost-effectiveness of a nonpharmacological intervention in pediatric burn care. Value in health. 2015;18(5):631-7.

51. Brown NJ, Kimble RM, Rodger S, Ware RS, Cuttle L. Play and heal: randomized controlled trial of Ditto™ intervention efficacy on improving re-epithelialization in pediatric burns. Burns. 2014;40(2):204-13.

52. Carr J, Crowley N. Prophylactic sequential bronchoscopy after inhalation injury: results from a three-year prospective randomized trial. European Journal of Trauma and Emergency Surgery. 2013;39(2):177-83.

53. Chahed J, Ksia A, Selmi W, Hidouri S, Sahnoun L, Krichene I, et al. Burns injury in children: is antibiotic prophylaxis recommended? African journal of paediatric surgery. 2014;11(4):323.

54. Chen G, Shao H, Pan X. A randomized controlled trial to compare the effects of liquid versus powdered recombinant human growth hormone in treating patients with severe burns. Biomedical reports. 2016;4(5):551-6.

55. Chen L, Meng K, Su W, Fu Y. The effect of continuous sedation therapy on immunomodulation, plasma levels of antioxidants, and indicators of tissue repair in post-burn sepsis patients. Cell biochemistry and biophysics. 2015;73(2):473-8.

56. Chen X, Feng X, Xie J, Ruan S, Lin Y, Lin Z, et al. Application of acellular dermal xenografts in full-thickness skin burns. Experimental and therapeutic medicine. 2013;6(1):194-8.

57. Chester SJ, Stockton K, De Young A, Kipping B, Tyack Z, Griffin B, et al. Effectiveness of medical hypnosis for pain reduction and faster wound healing in pediatric acute burn injury: study protocol for a randomized controlled trial. Trials. 2016;17(1):223.

58. Chi Y, Chai J, Luo H, Zhang Q, Feng R. Safety of recombinant human granulocyte-macrophage colony-stimulating factor in healing pediatric severe burns. Genet Mol Res. 2015;14(1):2735-41.

59. Cho YS, Choi YH. Comparison of three cooling methods for burn patients: A randomized clinical trial. Burns. 2017;43(3):502-8.

60. Cho YS, Jeon JH, Hong A, Yang HT, Yim H, Cho YS, et al. The effect of burn rehabilitation massage therapy on hypertrophic scar after burn: a randomized controlled trial. Burns. 2014;40(8):1513-20.

61. Cho YS, Joo SY, Cui H, Cho S-R, Yim H, Seo CH. Effect of extracorporeal shock wave therapy on scar pain in burn patients: a prospective, randomized, single-blind, placebo-controlled study. Medicine. 2016;95(32).

62. Chong SJ, Kan EM, Song C, Soh CR, Lu J. Characterization of early thermal burns and the effects of hyperbaric oxygen treatment: a pilot study. Diving and hyperbaric medicine. 2013:157.

63. Clayton RP, Wurzer P, Andersen CR, Mlcak RP, Herndon DN, Suman OE. Effects of different duration exercise programs in children with severe burns. Burns. 2017;43(4):796-803.

64. Cone L, Gottschlich M, Khoury J, Simakajornboon N, Kagan R. The Effect of Healing Touch on Sleep Patterns of Pediatric Burn Patients: A Prospective Pilot Study. J Sleep Disor: Treat Care 3: 2. of. 2014;6:2.

65. Csontos C, Rezman B, Foldi V, Bogar L, Drenkovics L, Röth E, et al. Effect of N-acetylcysteine treatment on oxidative stress and inflammation after severe burn. Burns. 2012;38(3):428-37.

66. Cucereanu-Bădică I, Luca I, Negreș S, Grințescu I, Lascăr I. The effects of intravenous glutamine supplementation in severely burned multiple traumatized patients. Farmacia; 2013.

For peer review only - http://bmjopen.bmj.com/site/about/guidelines.xhtml

67. Donovan ML, Muller MJ, Simpson C, Rudd M, Paratz J. Interim pressure garment therapy (4–6 mmHg) and its effect on donor site healing in burn patients: study protocol for a randomised controlled trial. Trials. 2016;17(1):214.

68. Ebid AA, Ahmed MT, Eid MM, Mohamed MSE. Effect of whole body vibration on leg muscle strength after healed burns: a randomized controlled trial. Burns. 2012;38(7):1019-26.

69. Ebid AA, El-Shamy SM, Draz AH. Effect of isokinetic training on muscle strength, size and gait after healed pediatric burn: A randomized controlled study. Burns. 2014;40(1):97-105.

70. Ebid AA, Omar MT, El Baky AMA. Effect of 12-week isokinetic training on muscle strength in adult with healed thermal burn. Burns. 2012;38(1):61-8.

71. Edionwe J, Hess C, Fernandez-Rio J, Herndon DN, Andersen CR, Klein GL, et al. Effects of whole-body vibration exercise on bone mineral content and density in thermally injured children. Burns. 2016;42(3):605-13.

72. Ehmer AIR, Mh; Khan, M; Hasan, F; Khaliq, Ra; Zehra, S. Efficacy of platelet rich plasma application in comparison to conventional dressing therapy in partial thickness burn wound. Medical Forum Monthly [Internet]. 2014; 25(1):[27-30 pp.]. Available from: http://onlinelibrary.wiley.com/o/cochrane/clcentral/articles/602/CN-01012602/frame.html.

73. El-Ghazely M, Mahmoud W, Atia M, Eldip E. Effect of probiotic administration in the therapy of pediatric thermal burn. Annals of burns and fire disasters. 2016;29(4):268.

74. Elsharnouby NM, Eid HE, Elezz NFA, Aboelatta YA. Heparin/N-acetylcysteine: an adjuvant in the management of burn inhalation injury: a study of different doses. Journal of critical care. 2014;29(1):182. e1-. e4.

75. Fatemi MJ, Salehi H, Akbari H, Alinejad F, Saberi M, Mousavi SJ, et al. The effect of levamisole on mortality rate among patients with severe burn injuries. Journal of research in medical sciences: the official journal of Isfahan University of Medical Sciences. 2013;18(9):795.

76. Finnerty CC, Ali A, McLean J, Benjamin N, Clayton RP, Andersen CR, et al. Impact of stress-induced diabetes on outcomes in severely burned children. Journal of the American College of Surgeons. 2014;218(4):783-95.

77. Gardien KL, Marck RE, Bloemen MC, Waaijman T, Gibbs S, Ulrich MM, et al. Outcome of burns treated with autologous cultured proliferating epidermal cells: a prospective randomized multicenter intrapatient comparative trial. Cell transplantation. 2016;25(3):437-48.

78. Kee EG, Kimble R, Cuttle L, Khan A, Stockton K. Randomized controlled trial of three burns dressings for partial thickness burns in children. Burns. 2015;41(5):946-55.

79. Genuino GAS, Baluyut-Angeles KV, Espiritu APT, Lapitan MCM, Buckley BS. Topical petrolatum gel alone versus topical silver sulfadiazine with standard gauze dressings for the treatment of superficial partial thickness burns in adults: a randomized controlled trial. Burns. 2014;40(7):1267-73.

80. Glas GJ, Muller J, Binnekade JM, Cleffken B, Colpaert K, Dixon B, et al. HEPBURN-investigating the efficacy and safety of nebulized heparin versus placebo in burn patients with inhalation trauma: study protocol for a multi-center randomized controlled trial. Trials. 2014;15(1):91.

81. Zhang Y, Gu F, Wang F, Zhang Y. Effects of early enteral nutrition on the gastrointestinal motility and intestinal mucosal barrier of patients with burn-induced invasive fungal infection. Pakistan journal of medical sciences. 2016;32(3):599.

For peer review only - http://bmjopen.bmj.com/site/about/guidelines.xhtml

82. Günter CI, Bader A, Dornseifer U, Egert S, Dunda S, Grieb G, et al. A multi-center study on the regenerative effects of erythropoietin in burn and scalding injuries: study protocol for a randomized controlled trial. Trials. 2013;14(1):124.

83. Hardee JP, Porter C, Sidossis LS, Børsheim E, Carson JA, Herndon DN, et al. Early rehabilitative exercise training in the recovery from pediatric burn. Medicine and science in sports and exercise. 2014;46(9):1710.

84. Hayashida K, Akita S. Quality of pediatric second-degree burn wound scars following the application of basic fibroblast growth factor: results of a randomized, controlled pilot study. Ostomy-Wound Management. 2012;58(8):32.

85. Herndon DN, Rodriguez NA, Diaz EC, Hegde S, Jennings K, Mlcak RP, et al. Long-term propranolol use in severely burned pediatric patients: a randomized controlled study. Annals of surgery. 2012;256(3):402.

86. Herndon DN, Voigt CD, Capek KD, Wurzer P, Guillory A, Kline A, et al. Reversal of growth arrest with the combined administration of oxandrolone and propranolol in severely burned children. Annals of surgery. 2016;264(3):421.

87. Hop MJ, Stekelenburg CM, Hiddingh J, Kuipers HC, Middelkoop E, Nieuwenhuis MK, et al. Cost-effectiveness of laser doppler imaging in burn care in the Netherlands: a randomized controlled trial. Plastic and reconstructive surgery. 2016;137(1):166e-76e.

88. Hsu K-C, Chen LF, Hsiep PH. Effect of music intervention on burn patients' pain and anxiety during dressing changes. burns. 2016;42(8):1789-96.

89. Huang G, Liang B, Liu G, Liu K, Ding Z. Low dose of glucocorticoid decreases the incidence of complications in severely burned patients by attenuating systemic inflammation. Journal of critical care. 2015;30(2):436. e7-. e11.

90. Hyland EJ, D'Cruz R, Harvey JG, Moir J, Parkinson C, Holland AJ. An assessment of early Child Life Therapy pain and anxiety management: a prospective randomised controlled trial. Burns. 2015;41(8):1642-52.

91. Hyland EJ, D'Cruz R, Menon S, Chan Q, Harvey JG, Lawrence T, et al. Prospective, randomised controlled trial comparing Versajet™ hydrosurgery and conventional debridement of partial thickness paediatric burns. Burns. 2015;41(4):700-7.

92. Isaac K, Umraw N, Cartotto R. Construction of Skin Graft Seams in Burn Patients: A Prospective Randomized Double-Blinded Study. Journal of Burn Care & Research. 2016;37(6):397-403.

93. Jang KU, Choi JS, Mun JH, Jeon JH, Seo CH, Kim JH. Multi-axis shoulder abduction splint in acute burn rehabilitation: a randomized controlled pilot trial. Clinical rehabilitation. 2015;29(5):439-46.

94. Jeschke MG, Abdullahi A, Burnett M, Rehou S, Stanojcic M. Glucose control in severely burned patients using metformin: an interim safety and efficacy analysis of a phase II randomized controlled trial. Annals of surgery. 2016;264(3):518-27.

95. Jeschke MG, Williams FN, Finnerty CC, Rodriguez NA, Kulp GA, Ferrando A, et al. The effect of ketoconazole on post-burn inflammation, hypermetabolism and clinical outcomes. PloS one. 2012;7(5):e35465.

96. Kaheni S, Sadegh Rezai M, Bagheri-Nesami M, Goudarzian AH. The effect of Distraction Technique on the pain of Dressing change among 3-6 year-old children. International Journal of Pediatrics. 2016;4(4):1603-10.

97. Keorochana K, Chaiyasuk P, Muangman P. Prospective randomize-controlled comparison between silicone plus herbal extract gel versus Aloe vera gel for burn scar prophylaxis. Wound Medicine. 2015;9:1-4.

For peer review only - http://bmjopen.bmj.com/site/about/guidelines.xhtml

98. Kibor D, Nyaim O, Wanjeri K. Effects of Enteral Glutamine Supplementation on Reduction of Infection in Adult Patients with Severe Burns. East African medical journal. 2014;91(1):33-6.

99. Kim J-B, Cho YS, Jang KU, Joo SY, Choi JS, Seo CH. Effects of sustained release growth hormone treatment during the rehabilitation of adult severe burn survivors. Growth Hormone & IGF Research. 2016;27:1-6.

100. Kipping B, Rodger S, Miller K, Kimble RM. Virtual reality for acute pain reduction in adolescents undergoing burn wound care: a prospective randomized controlled trial. Burns. 2012;38(5):650-7.

101. Kolmus AM, Holland AE, Byrne MJ, Cleland HJ. The effects of splinting on shoulder function in adult burns. Burns. 2012;38(5):638-44.

102. Lagus H, Sarlomo-Rikala M, Böhling T, Vuola J. Prospective study on burns treated with Integra®, a cellulose sponge and split thickness skin graft: comparative clinical and histological study—randomized controlled trial. Burns. 2013;39(8):1577-87.

103. Lairet KF, Baer D, Leas ML, Renz EM, Cancio LC. Evaluation of an oxygen-diffusion dressing for accelerated healing of donor-site wounds. Journal of Burn Care & Research. 2014;35(3):214-8.

104. Lak S, Ostadrahimi A, Nagili B, Asghari-Jafarabadi M, Beigzali S, Salehi F, et al. Anti-Inflammatory effect of taurine in burned patients. Advanced pharmaceutical bulletin. 2015;5(4):531.

105. Lewis PA, Wright K, Webster A, Steer M, Rudd M, Doubrovsky A, et al. A randomized controlled pilot study comparing aqueous cream with a beeswax and herbal oil cream in the provision of relief from postburn pruritis. Journal of Burn Care & Research. 2012;33(4):e195-e200.

106. Li X, Meng X, Wang X, Li Y, Li W, Lv X, et al. Human acellular dermal matrix allograft: A randomized, controlled human trial for the long-term evaluation of patients with extensive burns. Burns. 2015;41(4):689-99.

107. López-Rodríguez L, Miguel A, García-Hierro P, Herrero R, Martins J, van Saene HK, et al. Selective digestive decontamination attenuates organ dysfunction in critically ill burn patients. Shock. 2016;46(5):492-7.

108. Lorello DJ, Peck M, Albrecht M, Richey KJ, Pressman MA. Results of a prospective randomized controlled trial of early ambulation for patients with lower extremity autografts. Journal of Burn Care & Research. 2014;35(5):431-6.

109. Mabrouk AB, Ns; Helal, Ha; Zaki, Bm; Maher, A. Moist occlusive dressing (Aquacel(®) Ag) versus moist open dressing (MEBO(®)) in the management of partial-thickness facial burns: a comparative study in Ain Shams University. Burns : journal of the International Society for Burn Injuries [Internet]. 2012; 38(3):[396-403 pp.]. Available from: http://onlinelibrary.wiley.com/o/cochrane/clcentral/articles/469/CN-00830469/frame.html.

110. Maghsoudi H, Nezami N, Mirzajanzadeh M. Enhancement of burn wounds healing by platelet dressing. International journal of burns and trauma. 2013;3(2):96.

111. Mahmoud W, Mostafa W, Abdel-Khalek A, Shalaby H. Effect of immune-enhancing diets on the outcomes of patients after major burns. Annals of burns and fire disasters. 2014;27(4):192.

112. Malin EW, Galin CM, Lairet KF, Huzar TF, Williams JF, Renz EM, et al. Silver-coated nylon dressing plus active DC microcurrent for healing of autogenous skin donor sites. Annals of plastic surgery. 2013;71(5):481-4.

113. Marck RE, Gardien KL, Stekelenburg CM, Vehmeijer M, Baas D, Tuinebreijer WE, et al. The application of platelet-rich plasma in the treatment of deep dermal burns: A randomized, double-blind, intra-patient controlled study. Wound Repair and Regeneration. 2016;24(4):712-20.

114. Mayes T, Gottschlich MM, James LE, Allgeier C, Weitz J, Kagan RJ. Clinical safety and efficacy of probiotic administration following burn injury. Journal of Burn Care & Research. 2015;36(1):92-9.

For peer review only - http://bmjopen.bmj.com/site/about/guidelines.xhtml

115. McBride CA, Kimble RM, Stockton K. Three donor site dressings in pediatric split-thickness skin grafts: study protocol for a randomised controlled trial. Trials. 2015;16(1):43.

116. Moghazy A, Adly O, Elbadawy M, Hashem R. Evaluation of who oral rehydration solution (ORS) and salt tablets in resuscitating adult patients with burns covering more than 15% of total body surface area (TBSA). Annals of burns and fire disasters. 2016;29(1):43.

117. Mohamed SA-R, Mohamed NN. Efficacy and adverse events of early high-frequency oscillatory ventilation in adult burn patients with acute respiratory distress syndrome. Egyptian Journal of Anaesthesia. 2016;32(3):421-9.

118. Mohammadi AA, Johari HG, Eskandari S. Effect of amniotic membrane on graft take in extremity burns. Burns. 2013;39(6):1137-41.

119. Moravvej H, Hormozi AK, Hosseini SN, Sorouri R, Mozafari N, Ghazisaidi MR, et al. Comparison of the application of allogeneic fibroblast and autologous mesh grafting with the conventional method in the treatment of third-degree burns. Journal of Burn Care & Research. 2016;37(1):e90-e5.

120. Mowafy ZM, Monem MA, Zoheiry IMI, Khowailed KA-E, Ahmed BR. Effect of Low-Intensity Laser on the Neuropathic Common Peroneal Nerve Post Burn.

121. Muangman P, Praditsuktavorn B, Chinaroonchai K, Chuntrasakul C. Clinical efficacy test of polyester containing herbal extract dressings in burn wound healing. The international journal of lower extremity wounds. 2016;15(3):203-12.

122. Dittrich MHM, de Carvalho WB, Lavado EL. Evaluation of the "early" use of albumin in children with extensive burns: a randomized controlled trial. Pediatric critical care medicine. 2016;17(6):e280-e6.

123. Najmi M, Shariatpanahi ZV, Tolouei M, Amiri Z. Effect of oral olive oil on healing of 10–20% total body surface area burn wounds in hospitalized patients. Burns. 2015;41(3):493-6.

124. Nedelec B, Rachelska G, Parnell LK, LaSalle L. Double-blind, randomized, pilot study assessing the resolution of postburn pruritus. Journal of Burn Care & Research. 2012;33(3):398-406.

125. Nikkhah D, Booth S, Tay S, Gilbert P, Dheansa B. Comparing outcomes of sheet grafting with 1: 1 mesh grafting in patients with thermal burns: a randomized trial. Burns. 2015;41(2):257-64.

126. Noorbala MT, Noorbala M, Dashti-Rahmatabadi MH, Noorbala M, Noorbala R, Mozaffary B. Comparison of Hydrogel Produced by Radiation as Applied at the Research Center (Yazd Branch) With MaxGel and Routine Dressing for Second-Degree Burn Repair in Yazd Burn Hospital. Iranian Red Crescent Medical Journal. 2016;18(8).

127. Oen IM, van Baar ME, Middelkoop E, Nieuwenhuis MK. Effectiveness of Cerium Nitrate–Silver Sulfadiazine in the Treatment of Facial Burns: A Multicenter, Randomized, Controlled Trial. Plastic and reconstructive surgery. 2012;130(2):274e-83e.

128. Omar MT, Hegazy FA, Mokashi SP. Influences of purposeful activity versus rote exercise on improving pain and hand function in pediatric burn. Burns. 2012;38(2):261-8.

129. Orrey DC, Halawa OI, Bortsov AV, Shupp JW, Jones SW, Haith LR, et al. Results of a pilot multi-center genotype-based randomized placebo-controlled trial of propranolol to reduce pain after major thermal burn injury. The Clinical journal of pain. 2015;31(1):21.

130. Omranifard M, Heidari M, Farajzadegan Z, Niktabar MR, Motamedi N. Botulinum Toxin and Burn Induces Contracture. Archives of plastic surgery. 2016;43(6):609-11.

For peer review only

For peer review only - http://bmjopen.bmj.com/site/about/guidelines.xhtml

131. Ostadrahimi A, Nagili B, Asghari-Jafarabadi M, Beigzali S, Zalouli H, Lak S. A Proper Enteral Nutrition Support Improves Sequential Organ Failure Score and Decreases Length of Stay in Hospital in Burned Patients. Iranian Red Crescent Medical Journal. 2016;18(2).

132. Ostlie DJ, Juang D, Aguayo P, Pettiford-Cunningham JP, Erkmann EA, Rash DE, et al. Topical silver sulfadiazine vs collagenase ointment for the treatment of partial thickness burns in children: a prospective randomized trial. Journal of pediatric surgery. 2012;47(6):1204-7.

133. Ottomann C, Stojadinovic A, Lavin PT, Gannon FH, Heggeness MH, Thiele R, et al. Prospective randomized phase II trial of accelerated reepithelialization of superficial second-degree burn wounds using extracorporeal shock wave therapy. Annals of surgery. 2012;255(1):23-9.

134. Ouyang J, Chen Y-c, Luo G-x, Yan H, Peng Y-z, Huang Y-s, et al. A randomized and controlled multicenter prospective study of the chinese medicinal compound fufang xuelian burn ointment for the treatment of superficial and deep second-degree burn wounds. Cell biochemistry and biophysics. 2014;69(3):467-74.

135. Palmieri TL, Greenhalgh DG, Sen S. Prospective comparison of packed red blood cell–to–fresh frozen plasma transfusion ratio of 4: 1 versus 1: 1 during acute massive burn excision. Journal of Trauma and Acute Care Surgery. 2013;74(1):76-83.

136. Panahi Y, Beiraghdar F, Akbari H, Bekhradi H, Taghizadeh M, Sahebkar A. A herbal cream consisting of Aloe vera, Lavandula stoechas, and Pelargonium roseum as an alternative for silver sulfadiazine in burn management. Asian Biomedicine. 2012;6(2):273-8.

137. Parker M, Delahunty B, Heberlein N, Devenish N, Wood FM, Jackson T, et al. Interactive gaming consoles reduced pain during acute minor burn rehabilitation: a randomized, pilot trial. Burns. 2016;42(1):91-6.

138. Parry I, Painting L, Bagley A, Kawada J, Molitor F, Sen S, et al. A pilot prospective randomized control trial comparing exercises using videogame therapy to standard physical therapy: 6 months follow-up. Journal of Burn Care & Research. 2015;36(5):534-44.

139. Patton ML, Mullins RF, Smith D, Korentager R. An open, prospective, randomized pilot investigation evaluating pain with the use of a soft silicone wound contact layer vs bridal veil and staples on split thickness skin grafts as a primary dressing. Journal of Burn Care & Research. 2013;34(6):674-81.

140. Peña R, Ramirez LL, Crandall CG, Wolf SE, Herndon DN, Suman OE. Effects of community-based exercise in children with severe burns: a randomized trial. Burns. 2016;42(1):41-7.

141. Porro LJ, Al-Mousawi AM, Williams F, Herndon DN, Mlcak RP, Suman OE. Effects of propranolol and exercise training in children with severe burns. The Journal of pediatrics. 2013;162(4):799-803. e1.

142. Porro LJ, Herndon DN, Rodriguez NA, Jennings K, Klein GL, Mlcak RP, et al. Five-year outcomes after oxandrolone administration in severely burned children: a randomized clinical trial of safety and efficacy. Journal of the American College of Surgeons. 2012;214(4):489-502.

143. Santos Portilla A, Bravo GL, Miraval FK, Villamar MF, Schneider JC, Ryan CM, et al. A feasibility study assessing cortical plasticity in chronic neuropathic pain following burn injury. Journal of Burn Care & Research. 2013;34(1):e48-e52.

144. Rashaan ZM, Krijnen P, van den Akker-van ME, van Baar ME, Vloemans AF, Dokter J, et al. Clinical effectiveness, quality of life and cost-effectiveness of Flaminal® versus Flamazine® in the treatment of partial thickness burns: study protocol for a randomized controlled trial. Trials. 2016;17(1):122.

145. Reeves PT, Herndon DN, Tanksley JD, Jennings K, Klein GL, Mlcak RP, et al. Five-year outcomes after long-term oxandrolone administration in severely burned children: a randomized clinical trial. Shock (Augusta, Ga). 2016;45(4):367.

For peer review only

For peer review only - http://bmjopen.bmj.com/site/about/guidelines.xhtml

146. Rosenberg L, Krieger Y, Bogdanov-Berezovski A, Silberstein E, Shoham Y, Singer AJ. A novel rapid and selective enzymatic debridement agent for burn wound management: a multi-center RCT. Burns. 2014;40(3):466-74.

147. Rousseau A-F, Foidart-Desalle M, Ledoux D, Remy C, Croisier J-L, Damas P, et al. Effects of cholecalciferol supplementation and optimized calcium intakes on vitamin D status, muscle strength and bone health: a one-year pilot randomized controlled trial in adults with severe burns. Burns. 2015;41(2):317-25.

148. Salehi SH, As'adi K, Mousavi SJ, Shoar S. Evaluation of amniotic membrane effectiveness in skin graft donor site dressing in burn patients. Indian Journal of Surgery. 2015;77(2):427-31.

149. Schaden E, Kimberger O, Kraincuk P, Baron D, Metnitz P, Kozek-Langenecker S. Perioperative treatment algorithm for bleeding burn patients reduces allogeneic blood product requirements. British journal of anaesthesia. 2012;109(3):376-81.

150. Schulz A, Depner C, Lefering R, Kricheldorff J, Kästner S, Fuchs PC, et al. A prospective clinical trial comparing Biobrane® Dressilk® and PolyMem® dressings on partial-thickness skin graft donor sites. Burns. 2016;42(2):345-55.

151. Shahzad MN, Ahmed N. Effectiveness of Aloe vera gel compared with 1% silver sulphadiazine cream as burn wound dressing in second degree burns. J Pak Med Assoc. 2013;63(2):225-30.

152. Siadat AH, Iraji F, Bahrami R, Nilfroushzadeh MA, Asilian A, Shariat S, et al. The comparison between modified kligman formulation versus kligman formulation and intense pulsed light in the treatment of the post-burn hyperpigmentation. Advanced biomedical research. 2016;5.

153. Dallal MS, Safdari R, Koochak HE, Sharifi-Yazdi S, Akhoondinasab M, Pourmand M, et al. A comparison between occlusive and exposure dressing in the management of burn wound. Burns. 2016;42(3):578-82.

154. Sood R, Roggy DE, Zieger MJ, Nazim M, Hartman BC, Gibbs JT. A comparative study of spray keratinocytes and autologous meshed split-thickness skin graft in the treatment of acute burn injuries. Wounds: a compendium of clinical research and practice. 2015;27(2):31-40.

155. Sousse LE, Herndon DN, Mlcak RP, Lee JO, Andersen CR, Zovath AJ, et al. Long-term administration of oxandrolone improves lung function in pediatric burned patients. Journal of Burn Care & Research. 2016;37(5):273-7.

156. Tang H, Lv G, Fu J, Niu X, Li Y, Zhang M, et al. An open, parallel, randomized, comparative, multicenter investigation evaluating the efficacy and tolerability of Mepilex Ag versus silver sulfadiazine in the treatment of deep partial-thickness burn injuries. Journal of Trauma and Acute Care Surgery. 2015;78(5):1000-7.

157. Taudorf EH, Danielsen PL, Paulsen IF, Togsverd-Bo K, Dierickx C, Paasch U, et al. Non-ablative fractional laser provides long-term improvement of mature burn scars–A randomized controlled trial with histological assessment. Lasers in surgery and medicine. 2015;47(2):141-7.

158. Tokarik M, Sjöberg F, Balik M, Pafcuga I, Broz L. Fluid therapy LiDCO controlled trial—optimization of volume resuscitation of extensively burned patients through noninvasive continuous real-time hemodynamic monitoring LiDCO. Journal of Burn Care & Research. 2013;34(5):537-42.

159. Vejdan SA, Khosravi M, Zojaji F. Burn Donor Site Dressing Using Melolin and Flexigrid Versus Conventional Dressing. Shiraz E-Medical Journal. 2015;16(1).

160. Venclauskiene A, Basevicius A, Zacharevskij E, Vaicekauskas V, Rimdeika R, Lukosevicius S. Laser Doppler imaging as a tool in the burn wound treatment protocol. Videosurgery and Other Miniinvasive Techniques. 2014;9(1):24.

For peer review only - http://bmjopen.bmj.com/site/about/guidelines.xhtml

161. Venet F, Plassais J, Textoris J, Cazalis M-A, Pachot A, Bertin-Maghit M, et al. Low-dose hydrocortisone reduces norepinephrine duration in severe burn patients: a randomized clinical trial. Critical Care. 2015;19(1):21.

162. Venkatachalapathy T. A comparative study of paediatric thermal burns treated with topical heparin and without heparin. Indian Journal of Surgery. 2014;76(4):282-7.

163. Verhaegen PD, Bloemen MC, van der Wal MB, Vloemans AF, Tempelman FR, Beerthuizen GI, et al. Skin stretching for primary closure of acute burn wounds. Burns. 2014;40(8):1727-37.

164. Voon K, Silberstein I, Eranki A, Phillips M, Wood FM, Edgar DW. Xbox Kinect™ based rehabilitation as a feasible adjunct for minor upper limb burns rehabilitation: A pilot RCT. Burns. 2016;42(8):1797-804.

165. Wang C, Wang J, Feng J. Local application of low-dose insulin in improving wound healing after deep burn surgery. Experimental and therapeutic medicine. 2016;12(4):2527-30.

166. Wibbenmeyer L, Eid A, Liao J, Heard J, Horsfield A, Kral L, et al. Gabapentin is ineffective as an analgesic adjunct in the immediate postburn period. Journal of Burn Care & Research. 2014;35(2):136-42.

167. Wood F, Martin L, Lewis D, Rawlins J, McWilliams T, Burrows S, et al. A prospective randomised clinical pilot study to compare the effectiveness of Biobrane® synthetic wound dressing, with or without autologous cell suspension, to the local standard treatment regimen in paediatric scald injuries. Burns. 2012;38(6):830-9.

168. Wu J, Xu R, Zhan R, Luo G, Niu X, Liu Y, et al. Effective symptomatic treatment for severe and intractable pruritus associated with severe burn-induced hypertrophic scars: A prospective, multicenter, controlled trial. Burns. 2016;42(5):1059-66.

169. Wurzer P, Branski LK, Clayton RP, Hundeshagen G, Forbes AA, Voigt CD, et al. Propranolol reduces cardiac index but does not adversely affect peripheral perfusion in severely burned children. Shock (Augusta, Ga). 2016;46(5):486.

170. Xiao H, Li C, Zhou X, Wang X, Wu Z, Zhang L, et al. A new method of microskin autografting with a Vaseline-based moisture dressing on granulation tissue. Burns. 2014;40(2):337-46.

171. Yan H, Chen J, Peng X. Recombinant human granulocyte-macrophage colony-stimulating factor hydrogel promotes healing of deep partial thickness burn wounds. Burns. 2012;38(6):877-81.

172. Yarboro DD. A comparative study of the dressings silver sulfadiazine and Aquacel Ag in the management of superficial partial-thickness burns. Advances in skin & wound care. 2013;26(6):259-62.

173. Yim H, Yang H-T, Cho Y-S, Kim D, Kim J-H, Chun W, et al. A clinical trial designed to evaluate the safety and effectiveness of a thermosensitive hydrogel-type cultured epidermal allograft for deep second-degree burns. Burns. 2014;40(8):1642-9.

174. Yuan L, Minghua C, Feifei D, Runxiu W, Ziqiang L, Chengyue M, et al. Study of the use of recombinant human granulocyte-macrophage colony-stimulating factor hydrogel externally to treat residual wounds of extensive deep partial-thickness burn. Burns. 2015;41(5):1086-91.

175. Zahmatkesh M, Manesh MJ, Babashahabi R. Effect of Olea ointment and Acetate Mafenide on burn wounds–A randomized clinical trial. Iranian journal of nursing and midwifery research. 2015;20(5):599.

176. Zhang J, Li X, Gao Y, Guo G, Xu C, Li G, et al. Effects of puerarin on the inflammatory role of burn-related procedural pain mediated by P2X7 receptors. Burns. 2013;39(4):610-8.

177. Zidan SM, Eleowa SA, Nasef MA, Abd-Almoktader MA, Elbatawy AM, Borhamy AG, et al. Maximizing the safety of glycerol preserved human amniotic membrane as a biological dressing. Burns. 2015;41(7):1498-503.

For peer review only - http://bmjopen.bmj.com/site/about/guidelines.xhtml

178. Zu H, Li Q, Huang P, Wang X. Therapeutic value of blood purification and prognostic utilities of early serum procalcitonin, C reactive protein, and brain natriuretic peptide levels in severely burned patients with sepsis. Cell biochemistry and biophysics. 2015;72(1):259-63.

179. Wasiak J, Cleland H, Campbell F. Dressings for superficial and partial thickness burns. Cochrane Database Syst Rev. 2008;4.

180. Clare G, Suleman H, Bunce C, Dua H. Amniotic membrane transplantation for acute ocular burns. Cochrane Database of Systematic Reviews. 2011;9.

181. Breederveld RS, Tuinebreijer WE. Recombinant human growth hormone for treating burns and donor sites. The Cochrane Library. 2014.

182. Dumville JC, Munson C. Negative pressure wound therapy for partial-thickness burns. Cochrane Database Syst Rev. 2012;12.

183. Barajas-Nava LA, Lopez-Alcalde J, Roqué i Figuls M, Solà I, Bonfill Cosp X. Antibiotic prophylaxis for preventing burn wound infection. Cochrane Database of Systematic Reviews. 2013;6.

184. Hoogewerf CJ, Van Baar ME, Hop MJ, Nieuwenhuis MK, Oen IM, Middelkoop E. Topical treatment for facial burns. The Cochrane Library. 2013.

185. Wasiak J, Mahar PD, McGuinness SK, Spinks A, Danilla S, Cleland H, et al. Intravenous lidocaine for the treatment of background or procedural burn pain. The Cochrane Library. 2014.

186. Tan HB, Danilla S, Murray A, Serra R, El Dib R, Henderson TO, et al. Immunonutrition as an adjuvant therapy for burns. The Cochrane Library. 2014.

187. Masters B, Wood F, Tuckerman J, Peng L. High carbohydrate, high protein, low fat versus low carbohydrate, high protein, high fat enteral feeds for burns. status and date: Edited (no change to conclusions), published in. 2008(3).

188. Potter S, Brigic A, Whiting P, Cawthorn S, Avery K, Donovan J, et al. Reporting clinical outcomes of breast reconstruction: a systematic review. Journal of the National Cancer Institute. 2010;103(1):31-46.

189. Blencowe NS, Strong S, McNair AG, Brookes ST, Crosby T, Griffin SM, et al. Reporting of short-term clinical outcomes after esophagectomy: a systematic review. Ann Surg. 2012;255(4):658-66.

190. Whitehead L, Perkins G, Clarey A, Haywood K. A systematic review of the outcomes reported in cardiac arrest clinical trials: the need for a core outcome set. Resuscitation. 2015;88:150-7.

191. Hayes J, Black N, Jenkinson C, Young J, Rowan K, Daly K, et al. Outcome measures for adult critical care: a systematic review. Health technology assessment (Winchester, England). 2000;4(24):1-111.

192. Zakrzewska JM, Lopez BC. Quality of reporting in evaluations of surgical treatment of trigeminal neuralgia: recommendations for future reports. Neurosurgery. 2003;53(1):110-22.

193. Riddle DL, Stratford PW, Bowman DH. Findings of extensive variation in the types of outcome measures used in hip and knee replacement clinical trials: a systematic review. Arthritis care & research. 2008;59(6):876-83.

194. McNaughton-Collins M, Walker-Corkery E, Barry MJ. Health-related quality of life, satisfaction, and economic outcome measures in studies of prostate cancer screening and treatment, 1990-2000. JNCI Monographs. 2004;2004(33):78-101.

For peer review only

For peer review only - http://bmjopen.bmj.com/site/about/guidelines.xhtml

195. Deyo RA, Battie M, Beurskens A, Bombardier C, Croft P, Koes B, et al. Outcome measures for low back pain research: a proposal for standardized use. Spine. 1998;23(18):2003-13.

196. Bruce J, Russell E, Mollison J, Krukowski Z. The quality of measurement of surgical wound infection as the basis for monitoring: a systematic review. Journal of Hospital Infection. 2001;49(2):99-108.

197. Bruce J, Krukowski ZH, Al-Khairy G, Russell EM, Park K. Systematic review of the definition and measurement of anastomotic leak after gastrointestinal surgery. British Journal of Surgery. 2001;88(9):1157-68.

198. Dodd S, Clarke M, Becker L, Mavergames C, Fish R, Williamson PR. A taxonomy has been developed for outcomes in medical research to help improve knowledge discovery. Journal of clinical epidemiology. 2017.

199. Boers M, Kirwan JR, Wells G, Beaton D, Gossec L, d'Agostino M-A, et al. Developing core outcome measurement sets for clinical trials: OMERACT filter 2.0. Journal of clinical epidemiology. 2014;67(7):745-53.

200. Clarke M. Standardising outcomes for clinical trials and systematic reviews. Trials. 2007;8(1):39.

201. Clarke M. Standardising outcomes in paediatric clinical trials. PLoS Medicine. 2008;5(4):e102.

202. Kirkham JJ, Boers M, Tugwell P, Clarke M, Williamson PR. Outcome measures in rheumatoid arthritis randomised trials over the last 50 years. Trials. 2013;14(1):324.

203. Bautista-Molano W, Navarro-Compán V, Landewé RB, Boers M, Kirkham JJ, van der Heijde D. How well are the ASAS/OMERACT Core Outcome Sets for Ankylosing Spondylitis implemented in randomized clinical trials? A systematic literature review. Clinical rheumatology. 2014;33(9):1313-22.

204. Falder S, Browne A, Edgar D, Staples E, Fong J, Rea S, et al. Core outcomes for adult burn survivors: a clinical overview. Burns. 2009;35(5):618-41.

205. Kirkham JJ, Davis K, Altman DG, Blazeby JM, Clarke M, Tunis S, et al. Core Outcome Set-STAndards for Development: The COS-STAD recommendations. PLoS medicine. 2017;14(11):e1002447.

206. Kirkham JJ, Gorst S, Altman DG, Blazeby JM, Clarke M, Devane D, et al. Core outcome set–STAndards for reporting: the COS-STAR statement. PLoS medicine. 2016;13(10):e1002148.

207. (COMET) COMiET.

208. Gargon E, Williamson PR, Altman DG, Blazeby JM, Clarke M. The COMET Initiative database: progress and activities from 2011 to 2013. Trials. 2014;15(1):279.

209. Chiarotto A, Ostelo RW, Turk DC, Buchbinder R, Boers M. Core outcome sets for research and clinical practice. Brazilian journal of physical therapy. 2017;21(2):77-84.

210. Gorst SL, Gargon E, Clarke M, Blazeby JM, Altman DG, Williamson PR. Choosing important health outcomes for comparative effectiveness research: an updated review and user survey. PLoS One. 2016;11(1):e0146444.

211. Biggane AM, Brading L, Ravaud P, Young B, Williamson PR. Survey indicated that core outcome set development is increasingly including patients, being conducted internationally and using Delphi surveys. Trials. 2018;19(1):113.

For peer review only - http://bmjopen.bmj.com/site/about/guidelines.xhtml

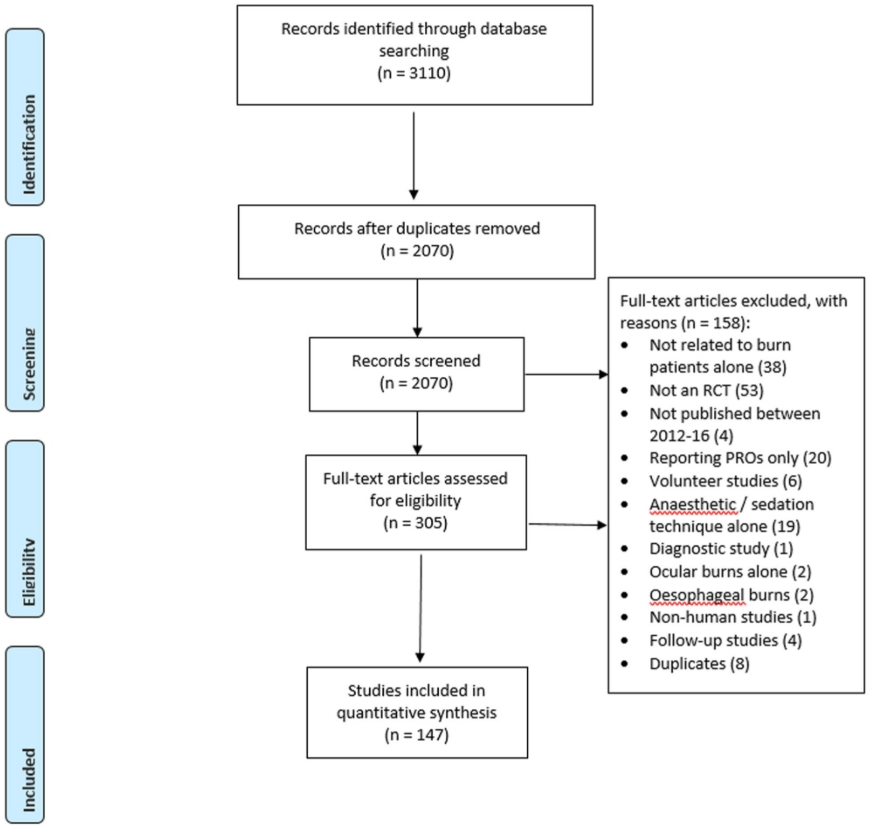

Figure 1: Prisma Flow Chart

174x228mm (300 x 300 DPI)

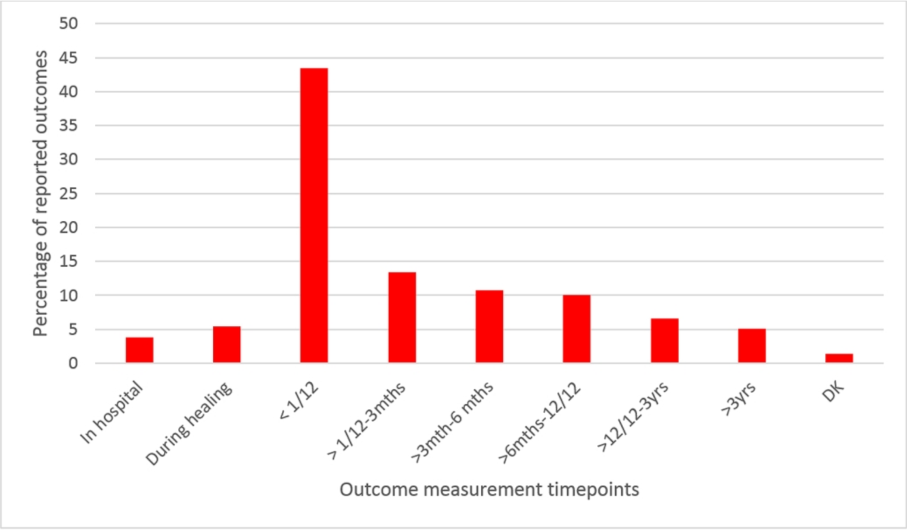

Figure 2: Reported timepoint at which outcome was assessed.

154x90mm (300 x 300 DPI)

Appendix A

Search Strategy using Ovid MEDLINE:

1. exp Burns/
2. burn*.tw.
3. scald*.tw.
4. (thermal* adj injur*).mp. [mp=title, abstract, original title, name of substance word, subject heading word, keyword heading word, protocol supplementary concept word, rare disease supplementary concept word, unique identifier, synonyms]
5. (smoke adj inhalation).mp. [mp=title, abstract, original title, name of substance word, subject heading word, keyword heading word, protocol supplementary concept word, rare disease supplementary concept word, unique identifier, synonyms]
6. 1 or 2 or 3 or 4 or 5
7. heartburn.mp. [mp=title, abstract, original title, name of substance word, subject heading word, keyword heading word, protocol supplementary concept word, rare disease supplementary concept word, unique identifier, synonyms]
8. burnout.mp. [mp=title, abstract, original title, name of substance word, subject heading word, keyword heading word, protocol supplementary concept word, rare disease supplementary concept word, unique identifier, synonyms]
9. (burn* adj out).mp. [mp=title, abstract, original title, name of substance word, subject heading word, keyword heading word, protocol supplementary concept word, rare disease supplementary concept word, unique identifier, synonyms]
10. burning.mp. [mp=title, abstract, original title, name of substance word, subject heading word, keyword heading word, protocol supplementary concept word, rare disease supplementary concept word, unique identifier, synonyms]
11. burnetii.mp. [mp=title, abstract, original title, name of substance word, subject heading word, keyword heading word, protocol supplementary concept word, rare disease supplementary concept word, unique identifier, synonyms]
12. burnish*.mp. [mp=title, abstract, original title, name of substance word, subject heading word, keyword heading word, protocol supplementary concept word, rare disease supplementary concept word, unique identifier, synonyms]
13. burnet*.mp. [mp=title, abstract, original title, name of substance word, subject heading word, keyword heading word, protocol supplementary concept word, rare disease supplementary concept word, unique identifier, synonyms]
14. 7 or 8 or 9 or 10 or 11 or 12 or 13
15. 6 not 14
16. (randomi?ed adj control* adj trial*).mp. [mp=title, abstract, original title, name of substance word, subject heading word, keyword heading word, protocol supplementary concept word, rare disease supplementary concept word, unique identifier, synonyms]
17. RCT.tw.
18. trial.tw.
19. 16 or 17 or 18
20. 15 and 19
21. limit 20 to (english language and humans and yr="2012 - 2016")
22. limit 21 to randomized controlled trial

For peer review only

PRISMA 2009 Checklist

Section/topic	#	Checklist item	Reported on page #
**TITLE**			
Title	1	Identify the report as a systematic review, meta-analysis, or both.	1
**ABSTRACT**			
Structured summary	2	Provide a structured summary including, as applicable: background; objectives; data sources; study eligibility criteria, participants, and interventions; study appraisal and synthesis methods; results; limitations; conclusions and implications of key findings; systematic review registration number.	1
**INTRODUCTION**			
Rationale	3	Describe the rationale for the review in the context of what is already known.	3
Objectives	4	Provide an explicit statement of questions being addressed with reference to participants, interventions, comparisons, outcomes, and study design (PICOS).	4
**METHODS**			
Protocol and registration	5	Indicate if a review protocol exists, if and where it can be accessed (e.g., Web address), and, if available, provide registration information including registration number.	4
Eligibility criteria	6	Specify study characteristics (e.g., PICOS, length of follow-up) and report characteristics (e.g., years considered, language, publication status) used as criteria for eligibility, giving rationale.	4,5
Information sources	7	Describe all information sources (e.g., databases with dates of coverage, contact with study authors to identify additional studies) in the search and date last searched.	4,5 Appendix A
Search	8	Present full electronic search strategy for at least one database, including any limits used, such that it could be repeated.	Appendix A
Study selection	9	State the process for selecting studies (i.e., screening, eligibility, included in systematic review, and, if applicable, included in the meta-analysis).	5
Data collection process	10	Describe method of data extraction from reports (e.g., piloted forms, independently, in duplicate) and any processes for obtaining and confirming data from investigators.	5,6
Data items	11	List and define all variables for which data were sought (e.g., PICOS, funding sources) and any assumptions and simplifications made.	5,6
Risk of bias in individual studies	12	Describe methods used for assessing risk of bias of individual studies (including specification of whether this was done at the study or outcome level), and how this information is to be used in any data synthesis.	Not done. Justification p5
Summary measures	13	State the principal summary measures (e.g., risk ratio, difference in means).	7,8

For peer review only

PRISMA 2009 Checklist

Synthesis of results	14	Describe the methods of handling data and combining results of studies, if done, including measures of consistency (e.g., I^2) for each meta-analysis.	Outcome data combined. P6,7

Section/topic	#	Checklist item	Reported on page #
Risk of bias across studies	15	Specify any assessment of risk of bias that may affect the cumulative evidence (e.g., publication bias, selective reporting within studies).	N/A
Additional analyses	16	Describe methods of additional analyses (e.g., sensitivity or subgroup analyses, meta-regression), if done, indicating which were pre-specified.	N/A
**RESULTS**			
Study selection	17	Give numbers of studies screened, assessed for eligibility, and included in the review, with reasons for exclusions at each stage, ideally with a flow diagram.	Figure 1, Table 2, p7
Study characteristics	18	For each study, present characteristics for which data were extracted (e.g., study size, PICOS, follow-up period) and provide the citations.	7,8
Risk of bias within studies	19	Present data on risk of bias of each study and, if available, any outcome level assessment (see item 12).	N/A
Results of individual studies	20	For all outcomes considered (benefits or harms), present, for each study: (a) simple summary data for each intervention group (b) effect estimates and confidence intervals, ideally with a forest plot.	N/A
Synthesis of results	21	Present results of each meta-analysis done, including confidence intervals and measures of consistency.	N/A
Risk of bias across studies	22	Present results of any assessment of risk of bias across studies (see Item 15).	Not done. Justification p5
Additional analysis	23	Give results of additional analyses, if done (e.g., sensitivity or subgroup analyses, meta-regression [see Item 16]).	N/A
**DISCUSSION**			
Summary of evidence	24	Summarize the main findings including the strength of evidence for each main outcome; consider their relevance to key groups (e.g., healthcare providers, users, and policy makers).	9
Limitations	25	Discuss limitations at study and outcome level (e.g., risk of bias), and at review-level (e.g., incomplete retrieval of identified research, reporting bias).	10
Conclusions	26	Provide a general interpretation of the results in the context of other evidence, and implications for future research.	9,10
**FUNDING**			

For peer review only - http://bmjopen.bmj.com/site/about/guidelines.xhtml

PRISMA 2009 Checklist

Funding	27	Describe sources of funding for the systematic review and other support (e.g., supply of data); role of funders for the systematic review.	11

From: Moher D, Liberati A, Tetzlaff J, Altman DG, The PRISMA Group (2009). Preferred Reporting Items for Systematic Reviews and Meta-Analyses: The PRISMA Statement. PLoS Med 6(7): e1000097. doi:10.1371/journal.pmed1000097

For more information, visit: **www.prisma-statement.org**.

For peer review only

For peer review only - http://bmjopen.bmj.com/site/about/guidelines.xhtml
